# Combining psychoanalytic concepts and computer science methodologies: an empirical study of the relationship between emotions and the Lacanian discourses

**DOI:** 10.3389/fpsyg.2026.1526215

**Published:** 2026-03-23

**Authors:** Minas Gadalla, Sotiris Nikoletseas, José Roberto de A. Amazonas

**Affiliations:** 1 Department of Computer Engineering and Informatics, University of Patras, Patras, Greece; 2 Department of Computer Engineering and Informatics, University of Patras and Computer Technology Institute and Press “Diophantus” (CTI), Patras, Greece; 3 Department of Computer Architecture, Technical University of Catalonia (UPC), Catalonia, Spain

**Keywords:** psychoanalysis, computer science, Natural Language Processing (NLP), Lacanian discourses, Lacanian Discourse Discovery (LDD), Lacanian Discourse Analysis (LDA), emotions

## Abstract

This research aimed to examine the interdisciplinary interaction between psychoanalysis and computer science, suggesting a mutually beneficial exchange. Indeed, psychoanalytic concepts can enrich technological applications that involve the human factor, such as social media and other interactive digital platforms. By providing deeper insights into the elusive, unconscious aspects of communication, psychoanalytic methods can enhance content-centric applications, including fake news detection and mental health diagnostics. Conversely, computer science, especially Artificial Intelligence (AI), can contribute quantitative concepts and methods to psychoanalysis, identifying patterns and emotional cues in human expression. In particular, this research aims to apply computer science methods to establish fundamental relationships between emotions and Lacanian discourses. These relationships are identified in our approach through empirical investigation and statistical analysis and ultimately validated through a theoretical (psychoanalytic) account. Notably, although emotions have been sporadically studied in Lacanian theory, a systematic, detailed investigation of their role is missing. Such a fine-grained understanding of the role of emotions can also make the identification of Lacanian discourses more effective and easier in practice. Our methods indicated the emotions with the highest differentiation power in the corresponding discourses; conversely, we identified for each discourse the most characteristic emotions it admits. We call this method Lacanian Discourse Discovery (LDD), and it simplifies (via systematizing) the identification of Lacanian discourses in texts. Although the main contribution of this study is inherently theoretical (psychoanalytic), it can also facilitate major practical applications in interactive digital systems. Indeed, our approach can be automated using Artificial Intelligence methods that effectively identify emotions (and corresponding discourses) in texts.

## 1 Introduction

This research aims to further explore the potential interactions between Psychoanalysis and Computer Science, envisioning a cross-fertilization that can be mutually beneficial for both fields. This exchange is expected to be advantageous in both “directions,” from Psychoanalysis to Computer Science and vice versa.

### 1.1 From psychoanalysis to computer science

We claim that psychoanalytic concepts and approaches can be highly instrumental in Computer Science applications that heavily involve the human factor, such as social media and other interactive digital platforms, systems, and tools. Indeed, in such text-based systems, psychoanalytic-based approaches have the potential to extract valuable, usually elusive information by providing insights into the underlying dynamics, motivations, and meanings of texts. They go beyond surface-level analysis and delve into the unconscious, possibly hidden aspects of communication. They can be applied to various types of texts and communication contexts, providing a deeper understanding of the message being conveyed and the mechanisms that shape the content. Such insight can significantly enhance the effectiveness and performance of diverse digital applications and systems that are inherently content-centric; such systems include software tools for detecting fake news on digital platforms, digital health applications for the early diagnosis of mental conditions and diseases, and software tools for deciding whether a given text is written by Artificial Intelligence (AI) programs or by humans.

### 1.2 From computer science to psychoanalysis

Sigmund Freud envisioned the future inclusion of quantitative, interdisciplinary concepts and methods (such as those from Physics) into psychoanalysis. In a similar spirit, Jacques Lacan expressed and visualized psychoanalytic concepts through analogs from combinatorial mathematics (sets and networks) and mathematical logic (logical formulae); in particular, he thought of the language of the unconscious as a chain of quasi-mathematical inscriptions, similar to a computer language as well as a cryptographic code. In this line, it is reasonable to assume that Computer Science methods can be quite helpful in establishing solid quantitative contributions to Psychoanalysis. In particular, the use of Machine Learning and more recently of the Large Language Models (LLMs) is high relevant, given its ability, when properly used, to identify complex or hidden patterns, emotional cues, and underlying themes in human speech or writing.

Regarding the first direction mentioned above, we have already conducted promising research on automated detection of fake news. In Gadalla et al. (2023, p. 2), we investigated the incorporation of human factors and user perception in text-based interactive media, focusing on how the reliability of user texts is influenced by behavioral and emotional dimensions. In particular, we designed a Psychoanalytic approach that uses Lacanian discourse types to capture and understand the underlying characteristics of texts (news headlines) and their inherent relation to real and fake news. The approach first identifies Lacanian discourses in a text (e.g., news headlines) and then uses an algorithm to predict whether the text is real or fake based on the type of Lacanian discourses present in it. The performance evaluation demonstrated high effectiveness and accuracy for this Psychoanalysis-based prediction compared with standard methods. As far as we know, this was the first time computational methods were systematically combined with Psychoanalysis.

Following up on this significant first step, the research discussed here addresses the “inverse direction,” applying Computer Science methodologies to Psychoanalysis. In particular, the purpose is to empirically investigate (and then theoretically validate) possible fundamental relations between emotions and Lacanian discourses. J. Lacan himself highlighted the significance of a few fundamental emotions (such as anxiety and anguish, Soler, 2016, p. 18). Recent research Bucci et al. (2022, p. 165) has investigated the important role of some emotions (affects) in Lacanian theory (Lacan, 2004d,h). However, there is a need to produce a study that systematically relates emotions with corresponding Lacanian discourses. This study aims to address this gap by empirically (statistically) investigating this relation in a systematic, fine-grained manner. Such a solid understanding of the relation between emotions and Lacanian discourses is important because it will make the identification of Lacanian discourses in texts easier and more robust, since it will be based on the (easier to grasp) presence of emotions in texts.

The method includes the following key steps: (a) the emotions in a given text are identified using a well-known, fine-grained set of 30 emotions; (b) the Lacanian discourses in the text are also identified; (c) a statistical investigation is performed to identify the potentially inherent relationship between emotions and Lacanian discourses: for each discourse, which are the most characteristic emotions it admits? Which emotions exhibit the highest differentiation power in terms of corresponding discourses? (d) finally, these statistical findings are theoretically (psychoanalytically) validated.

The main contribution of this research is a psychoanalytic one per se: the establishment of a systematic relationship among emotions and Lacanian discourses, and the introduction of the concept of Lacanian Discourse Discovery (LDD), which systematizes the discovery of the Lacanian discourses. Notably, this method can be automated to a great extent, since current computer-based methods (primarily employing AI systems and tools) can effectively detect emotions in texts. Thus, there is great potential to develop effective, real-world applications based on the automated identification of emotions and their corresponding discourse. It is important to emphasize that at this stage, this study does not have any diagnostic purpose.

After this Introduction, in Section 2.1, a theoretical framework is presented that includes how emotions appear in S. Freud's and J. Lacan's works, a review of the five Lacanian discourses, a description of the Lacanian Discourse Analysis (LDA), and a review of the classification of emotions as proposed by different authors. In Section 2.2, a brief presentation of related studies is provided. In Section 3, the adopted methodology is presented in detail. The results and the corresponding discussion are presented in Section 4. Section 5 concludes the paper, indicates future directions, and discusses potential applications of the achievements of this study.

## 2 Materials and methods

### 2.1 Theoretical framework

The objectives of this section are to review how emotions are treated in the research of S. Freud and J. Lacan, to review the five Lacanian discourses, to describe Lacanian Discourse Analysis (LDA), and to review the major proposed classification of emotions.

While S. Freud is universally recognized as the “father” of Psychoanalysis, it is worth briefly providing some information about J. Lacan's place in Psychoanalysis and how psychoanalysts and psychologists assess him.

J. Lacan (1901–1981) is considered one of the most influential and controversial figures in psychoanalytic theory. J. Lacan's study reinterpreted Freudian theory through the lenses of structural linguistics, topology, and philosophy, particularly drawing on Saussure, Levi-Strauss, and Hegel (Fink, 1996). J. Lacan's emphasis on language and the symbolic order marked a significant departure from the ego psychology dominant in mid-20th-century psychoanalysis (Evans, 1996, p. 162).

J. Lacan's position within psychoanalysis is both central and controversial. In France, Lacanian psychoanalysis has had and continues to have a significant institutional and clinical presence, influencing numerous schools of thought (Roudinesco, 1990, p. 25). However, in Anglo-American psychology, J. Lacan's study has often been marginalized for its abstract style and complex terminology (Leader, 2012, p. 81). His ideas have been influential in critical theory, feminist psychoanalysis, and cultural studies (Zizek, 2006).

Some psychoanalysts view J. Lacan's contributions as revitalizing the Freudian legacy and providing a richer conceptual framework for understanding subjectivity and language (Nobus, 1999, p. 102). Others criticize J. Lacan for a lack of clinical clarity or empirical support, raising concerns about the scientific rigor of Lacanian psychoanalysis (Crews, 1998, p. xxix). J. Lacan remains a major figure in psychoanalytic theory, yet his reception among psychologists and empirical researchers remains ambivalent.

#### 2.1.1 Emotions in Freud's works

In the literature in general, and in the psychoanalytic literature in particular, emotions and affects are often incorrectly used as synonyms of each other, or one is mistakenly used for the other.

It is not the purpose of this study to delve into the intricacies of each of these terms. This paper deals mainly with emotions. When the term affect appears, it indicates that this word was the original choice of the referenced author.

It is outside the scope of this study to present a comprehensive review of psychoanalytic theory. In fact, it is assumed that the reader is at least acquainted with its fundamental concepts, such as the Unconscious the triad (ego, id, and superego), and the theory of sexuality.

Since 1893, S. Freud, either alone or in collaboration with J. Breuer, in his research about Hysteria (Breuer and Freud, 1893–1895) and “The Neuropsychoses of Defence” (Freud, 1894), examined affects and their destination when submitted to the repression process. For example, in this latter research he stated (Freud, 1894, p. 303):

*For these patients whom I analyzed had enjoyed good mental health until an occurrence of incompatibility [Unverträglichkeit] took place in their ideational life–that is, until their ego [Ich] was faced with an experience, an idea, or a feeling that aroused such a **distressing affect** that the subject [Person] decided to forget about it because he had no confidence in his power to resolve the contradiction between that incompatible idea and his ego by means of thought activity*.

In the face of such incompatible ideas, patients try to “push the thing away,” make an effort not to think of it, or suppress it. When this kind of “forgetting” did not succeed, it led to various pathological reactions, producing either hysteria or an obsession or a hallucinatory psychosis. The memory trace and the **affect** which is attached to the idea are there once and for all and cannot be eradicated.

In 1915, S. Freud published his fundamental research on the Unconscious, in which he postulates the destination of the repressed **affect** (Freud, 1915b, p. 3002):

*The importance of the system Cs. (Pcs.) with regard to access to the release of affect and to action enables us to understand the part played by substitutive ideas in determining the form taken by illness. It is possible for the development of **affect** to proceed directly from the system Ucs.; in that case, the **affect** always has the character of anxiety **[anguish]**, for which all “repressed” **affects** are exchanged*.

in which Cs., Pcs., and Ucs stand for the Conscious, Preconscious, and Unconscious systems respectively.

In this study, we retain the term proposed in the Spanish version (Freud, 1915a), translated by Luis López Ballesteros y de Torres and formally approved by S. Freud, and will refer to **anguish** (instead of **anxiety**) as the result of repressed **affect**.

In summary, it may be said that:

- S. Freud uses the term **affect** to collectively refer to emotions;
- Emotions are essential elements of the etiology of neuropsychoses;
- Whether the emotions were repressed or not, along with the distressing experience, determines the development path of the neuropsychosis;
- No single emotion is identified as more important in the development of neuropsychoses;
- All repressed and undischarged emotions are exchanged for **anguish**.

#### 2.1.2 Emotions in Lacan's works

The *Vorstellungsrepräsentanzen* (the representatives of representations) are strictly equivalent to J. Lacan's signifiers. Affects are situated along the pleasure-unpleasure axis, are not completely repressed, have become disconnected from the original trauma, and can move among different *Vorstellungsrepräsentanzen*. Affects sliding from one representation to another lie (according to J. Lacan (Lacan 2004a), class given on November 26, 1958) about their origins. According to C. Soler in (Soler, 2016, p. 15):

*Placing at the beginning of mankind's fate the experience of an unmasterable excitation that overwhelms the subject and generates anguish that he qualifies as “real”, he bestows a very specific status on **anguish**: it is both effect and cause. It is the effect of a real encounter with the said excitation, but it is the cause of the repression that will generate symptoms and resonate in subsequent affects, first among which is “**anguish** as a signal”, which is both a memorial and a warning: a memorial of the first trauma and a warning about an imminent danger*.

To start with, Lacan (2004b, p. 65) posited that **anguish** is the affect that does not lie or deceive (*ne trompe pas*). It involves major bodily sensations, such as having a stone in one's throat or a heart racing too fast. **Anguish** has three characteristics:

1. there is a blurred threat;
2. it is experienced;
3. the subject knows that it concerns him/her, but has no explanation for it.

**Anguish** does not drift among signifiers; it remains attached to the original cause. It is not without an object; its object is the object *a* (*le petit a*), and it indicates the oncoming arrival of something that is real. Because it is tied to something real, it becomes an ally to interpretation.

Capitalism has replaced symbolic production with the objects it produces. People talk a great deal about the rise of depression in our era, but the true mood illness of capitalism is **anguish**. **Anguish** is the emotion tied to “subjective destitution;” it is an affect that arises when the subject perceives himself as an object. Scientific capitalism, with its technological effects, brings about destitution far more radically than psychoanalysis does: it uses and abuses subjects as instruments.

J. Lacan went far beyond the consideration of **anguish** by developing a complete “Theory of affects,” in which he states that no known affects lack a bodily component. Thus, to conceptualize affect, one must “include the body” (Lacan, 1974) and, as exposed in Soler (2016, p. 52):

The organic individual that supports the speaking subject represented by the signifier is not the body. There are:

1. the living organism, which is the object studied by biology and which psychoanalysts need to know little about;
2. the subject defined by his speech; and
3. the body of the subject, which is also studied by psychoanalysis since it is subject to symptoms.

J. Lacan presented an affect series on television that is both unique and surprising (Lacan, 1974).

According to Soler (2016, p. 84):

This series does not seek to cover all effects, but specifically those that are responses to the reality of the unconscious (*au réel de l'inconscient*)–in other words, to the impossible relationship between the sexes–and to its effects, which psychoanalysis alone illuminates.

.

.

.

From this vantage point, not all effects are equivalent. Of the four I have discussed, the first two (**sadness** and **joyful knowledge**) are related to knowledge, and the last two (**boredom** and **moroseness**) are related to sex. The series is thus organized as follows: on the one hand, as a function of one's ethical position in relation to knowledge, the difference between sadness and joyful knowledge; on the other hand, as a function of the historicity of discourse, with “our” boredom and “our” moroseness, the typical dominance of the latter corresponds to the effects of the unconscious on the body when the reparative semblances that nourished Eros are missing.

#### 2.1.3 The Lacanian discourses

J. Lacan proposed his four discourses in 1969 in his seminar L'envers de la psychanalyse (Lacan, 2004e, p. 11). He further developed his ideas in the following seminars as Lacan (2004g, p. 36) and Lacan (2004h, p. 91). His ideas were extremely well explained by Bailly (2009, p. 123–129).

The Four Discourses theory is a formalism for the different ways people relate to one another and for the economy of knowledge and enjoyment in social relationships. The general structure is represented by a *matheme*, as shown in Equation 1.


$$↑\frac{\mathrm{Agent}}{\text{Truth}}\vec{⤧}\frac{\text{other}}{\mathrm{Production}}↓$$ (1)

The transmitter of the discourse is the *Agent* occupying the top left position of the *matheme*. The *Agent* addresses the *other* occupying the top right position of the *matheme*. The *other* is the receiver of the message. This interaction is represented by the arrow pointing from the *Agent* to the *other*. What the *Agent* says is driven by the *Truth* hidden below the *Agent*, belonging to the Unconscious and not directly accessible. This driving is represented by the upward arrow pointing from the *Truth* to the *Agent*. The hidden *Truth* is embedded in the transmitted message. This is represented by the oblique arrow pointing from the *Truth* to the *other*. The message is interpreted by the *other*, both consciously and unconsciously, and a response, the *Production*, is delivered. The *Production* is the fourth element of the *matheme*, occupying the bottom right position of the *matheme*. The *Production* is delivered to the *Agent*, represented by the oblique arrow pointing from the *Production* to the *Agent*.

There is no arrow pointing from the *Production* to the *Truth*, indicating that the provided response is incomplete and unsatisfactory. Perfect communication between the transmitter and receiver sides is impossible. This impossibility arises because people are speaking beings and communication is constrained by language. Nevertheless, this impossibility is what creates and maintains the social bonds between beings.

The four elements of the *matheme* are placeholders for the roles assumed by the interacting parties in the communication process, namely:

- **S1**: the master signifier represents the true essence of the subject; it organizes both the psychical and everyday reality. It may be summarized by *who I am*.
- **S2**: represents the knowledge of the subject. It may be summarized by *what I know*.
- **a**: represents the object cause of desire. It may be summarized by *what I want*.
- **$**: represents the barred subject castrated by the language. It may be summarized by *what I speak*.

The four discourses are defined by the position each element occupies in the general *matheme* representation shown in Equation 1.

$↑\frac{\mathrm{S1}}{\text{\$}}\vec{⤧}\frac{\text{S2}}{a}↓$ **Master Discourse:** the *Agent* position is occupied by **S1** who addresses the *other* not as a person *per se* but as the holder of a knowledge (**S2**). The message may convey not only traces of authority and power but also the image the *Agent* builds about him/herself to be publicly disclosed. **S1** is driven by **$**, meaning that the message suffers from the limitations imposed by the language. The receiver **S2** cannot fully enjoy the possession of knowledge that is delivered to the *Agent* as the *Production* represented by **a**.

A counterclockwise rotation of the elements of the **Master Discourse** *matheme* leads to the second discourse.

$↑\frac{\mathrm{S1}}{\text{S2}}\vec{⤧}\frac{a}{\$}↓$ **University Discourse:** in this discourse **S1** is downgraded and becomes the hidden *Truth*. **S2** becomes the *Agent*. The transmitted message may convey verified and referenced information. The *Agent* may be a real expert or authority in his/her field of study, or can be just the false image of an expert whose expertise is derived from the supporting **S1**. It is a discourse that represents institutions and their manipulative actions upon the receivers of the message. The *Agent* addresses **a**, i.e., addresses the receiver's desire to be accepted by the prestigious **S1** and to incorporate some of its characteristics. The end result, i.e., the *Production*, is merely the receiver's submission to the transmitter.

A counterclockwise rotation of the elements of the **University Discourse** *matheme* leads to the third discourse.

$↑\frac{a}{\text{S2}}\vec{⤧}\frac{\$}{\mathrm{S1}}↓$ **Analyst Discourse:** in the discourse of the Analyst, the *Agent* becomes the **a** of the other, i.e., the other's desire to know more about him/herself. The *Agent* becomes the subject-supposed-to-know (**S2**) of the *Truth* about the other. The *Agent* addresses the other as a speaking being, employing a neutral curiosity, free of judgement, and aiming to provoke the other to generate a *Production* that reveals his/her master signifiers **S1**. This chain of signifiers may be interpreted and reflected back, as if by an empty mirror, to the other, who ends up knowing more about him/herself. In a long-term relationship, the other ends up learning that all knowledge acquired came from him/herself and that the *Agent* is no longer necessary.

Another counterclockwise rotation of the elements of the **Analyst Discourse** *matheme* leads to the final fourth discourse.

$↑\frac{\$}{a}\vec{⤧}\frac{\mathrm{S1}}{\mathrm{S2}}↓$ **Hysteric Discourse:** one does not have to be hysterical in the clinical sense to hold the discourse of the Hysteric; indeed, J. Lacan made it clear that this type of discourse in non-hysterical people is precisely what leads to true learning. The *Agent* as a speaking being **$**, driven by his/her desire to know **a**, addresses the *other* in his social role **S1** as someone that is able to produce true information and knowledge **S2**, the targeted *Production*. The *Production* is usually unsatisfactory, making the *Agent* continue the questioning till the knowledge **S2** of the *other* is exhausted.

In Seminar XVIII (Lacan, 2004f) and Seminar XIX (Lacan, 2004c, p. 66), J. Lacan reformulated the general structure of the discourse's *matheme*, as shown in Equation 2.


$$↑\frac{\mathrm{Semblance}}{\text{Truth}}\vec{⤧}\frac{\text{Jouissance}}{\mathrm{Surplus-jouissance}}↓$$ (2)

The *Agent* becomes the *Semblance* to indicate that the discourse is defined by the position or role someone takes in relation to the *other*. For example, the discourse of the **Master** takes shape when someone plays the role of the commanding agent. The position of the *other* is substituted by the *Jouissance* that is defined by Lacan (2004c, p. 66) in Seminar XIX as a disturbing dimension in the experience of the body. The subject is unable to experience itself as a self-sufficient enjoying entity. The enjoyment is conditioned by the addressing *Semblance* which is expected to manage it.

Last but not least, the *Production* of the discourse becomes the *Surplus-jouissance*. J. Lacan borrows Marx's concept of “surplus value” (Marx, 2018) to build the concept of “surplus-jouissance.” “Surplus value” is defined as the difference between the exchange value of products of labor (commodities) and the value that corresponds to the effort of producing these products, i.e., the means of production and labor power. Within the capitalist system, the sole objective is to extract surplus value; profit-making and capital expansion are the driving forces.

Lacan (2004d, p. 7) states that the general structure of discourse is homologous to Marx's system of capitalism. In capitalist production, surplus value and/or commodities are fetishized, whereas in discourse a fetishistic relation with surplus-jouissance is created.

In discourse, language is produced. The attempt to address jouissance through language produces a surplus of corporeal tension that lies beyond language itself. Such surplus-jouissance can only be located in the realm of fantasy or delusion.

By the end of 1960, J. Lacan began commenting on a fifth discourse, the **Capitalist Discourse**, highlighting its differences from the other four discourses. In 1972, in a lecture at the University of Milan (Contri, 1978, p. 32–55), J. Lacan presented the precise structure of the **Capitalist Discourse**, as shown in Equation 3.


$$↓\frac{\$}{\text{S}1}⤧\frac{\text{S}2}{a}↓$$ (3)

At first glance, the **Capitalist Discourse** appears to be a simple variation of the **Master Discourse**, but in fact it is a disruptive mutation of it. By comparing both structures, it is possible to identify three differences:

1. **$** and **S1** exchange places.
2. The arrow pointing upward on the left that makes the position of the truth inaccessible in the classic discourse changes into an arrow pointing downwards.
3. The horizontal arrow that established the connection between the *Agent* and the *other*, or between the *Semblance* and the *Jouissance* disappears.

In the **Master Discourse**, **S1** organizes the discourse due to its position as the *Agent* (*Semblance*). In the **Capitalist Discourse**, S1 has been degraded to occupy the Truth position that is not hidden anymore but has become accessible as indicated by the arrow pointing downwards on the transmitter's side. In the **Capitalist Discourse**, the castrated subject **$** does not express its needs or demands by addressing the *other*. **$**, by itself, elects a fetishized commodity to fulfill those needs or demands. The fetish materializes through the acquisition of an asset (**S2**) that most certainly does not meet the subject's expectations. This frustration fuels the *Production*, and the object-cause-of-desire **a** feeds back to **$**. The subject will try to combat this frustration by electing a new **S1**, and the cycle repeats itself.

The cycle **$** → **S1** → **S2** → **a** → **$** → …  can continue indefinitely. This cycle represents serious disorders such as addiction and compulsion. Addiction includes drugs, social media, pornography, and others. Compulsion includes buying, working, sex, and others.

The attachment to the **Capitalist Discourse** is driven by big corporations, the media, and marketing giants, who use all kinds of manipulation to enslave people to the carousel of consumerism. The Subject is reduced to a mere object to be used and discarded.

In the four classic discourses, social bonds are sustained by the impossibility of communication between the hidden *Truth* and the *Production*. In J. Lacan's terms, the non-rapport (▴) underpins the relationship between the *Agent* (*Semblance*) and the *other* (*Jouissance*).


$$\frac{\text{Agent}}{\text{Truth}}\vec{▴}\frac{\text{other}}{\text{Production}}$$

$$\frac{\text{Semblance}}{\text{Truth}}\vec{▴}\frac{\text{Jouissance}}{\text{Surplus-jouissance}}$$

In the **Capitalist Discourse** the social bonds are destroyed.

#### 2.1.4 Lacanian Discourses analysis and Lacanian Discourses Discovery

Lacanian Discourse Analysis (LDA) is not a system or methodology developed by Lacan himself but rather a way of applying Lacanian concepts to analyze texts. It is considered a subsequent scholarly effort, especially in the psychosocial domain, to create a “system” of text analysis aligned with Lacanian psychoanalytic theory, which was originally designed for therapeutic sessions. However, this paper does not aim to delve deeply into LDA or engage with the intricacies of psychoanalysis. Instead, it focuses on utilizing certain aspects of LDA, particularly one of the five Lacanian Discourses, for a specific application and goal. To reflect this distinction, we refer to this approach as *Lacanian Discourse Discovery* (LDD) rather than LDA.

The term “Discovery” is chosen deliberately to emphasize that this process of identifying the five discourses in a text does not involve analytical or methodological procedures, such as those outlined by Parker (2010, p. 156–172). Instead, the identification of the discourses is viewed as a discovery process, one that quickly and effectively uncovers the Lacanian discourses in a text without delving deeply into the text's attributes or the speakers' characteristics.

This work represents the first attempt to apply the Lacanian Discourses within the Natural Language Processing (NLP) domain of Computer Science; breaking down text or speech into smaller parts that computer programs can easily understand^1^ (Eisenstein, 2019).

The use of Lacanian approaches in text analysis is common in theoretical studies of philosophy and politics (Wetherell, 1999, p. 399–406), and in studies of structured texts (Parker, 2010, p. 156–172).

Structured text, such as a movie script written by a screenwriter for production and audience entertainment, has the advantage of allowing one to map the social, cultural, and situational factors influencing the text and the relationships between the characters. In the same way, this approach is applied to less structured texts. This is a new, radical approach to combining Lacanian psychoanalytic concepts in the NLP domain. This approach aims to quickly identify the five Lacanian discourses when there is no information about social or cultural factors, the gender or attributes of the subjects, or clear connections or interactions between the characters.

By bringing parts of Lacanian Discourse theory into the NLP domain, it enabled the achievement of:

1. An efficient method for discovering Lacanian discourses in short, unstructured texts such as everyday dialogues.
2. A foundation for the development of future computer-based applications using the Lacanian Discourses.
3. For the first time, the integration of Lacanian discourse concepts with NLP tasks in the field of Computer Science.

#### 2.1.5 Classification of emotions

The categorization of emotions is particularly complex and debated. Unlike more concrete categories (e.g., colors), emotions present unique challenges due to their subjective, multifaceted nature and linguistic constraints.

The relationship between language and emotion is far from straightforward. While words like “anger” or “fear” may appear to directly express internal states, psychological research shows that emotional words can serve multiple discursive functions from labeling to distancing or rhetorical emphasis (Barrett, 2006; Lindquist et al., 2015). The mere presence of an emotion term is not necessarily diagnostic of the speaker's felt emotion, especially in unconstrained everyday discourse (Wierzbicka, 1999).

In corpus linguistics and discourse psychology, scholars have analyzed how combinations of linguistic elements form patterned representations of power, ideology, or affect. For example, Ertel (1985) focused on the possibility that language might be a reservoir of cues signaling cognitive features underlying ideological commitment. In Mills (1985), the author states that:

Content analysis is the fundamental tool for analyzing and predicting the policies of communist states because: (1) survey research, archival studies, and firsthand observation of the national decision-making process are generally impractical, and (2) policies are often publicly justified because ideology is a more significant legitimizing factor than electoral victory, charisma, or heredity.

In Siderits et al. (1985), the authors examined how role and gender affect hostile and anxious communication. The role was manipulated using the Melian Dialogues, a technique borrowed from community organization training that asks participants to alternately assume the roles of superior and inferior power. A multivariate analysis of variance showed that while role had a highly significant effect (*p* < 0.0001) on the content of the communications, neither gender nor the order in which roles were assumed had a significant influence. The results were interpreted as a consequence of role justification.

Recent Polish research has examined how linguistic collocations reveal implicit conceptualizations of power (Citlak and Koziol, 2024). Similarly, the Linguistic Category Model (LCM), developed by Semin and Fiedler (1991) and Semin (2008), provides a systematic framework for analyzing the abstraction level of verbs and nouns in emotional or interpersonal language, thereby revealing subtle biases in attribution and social judgment.

Probably the most basic set of emotions found in human text comes from a Chinese encyclopedia compiled in the first century B.C.: What are the feelings of men? They are joy, anger, sadness, disliking, and liking. These five feelings belong to men without their having learned them (Chai and Chai, 1967, p. 379).

Such a basic scheme could be valid and used as an early-stage approach to identifying a state-of-the-art set of emotions for universal use. Progressively, the most well-known schema are Ekman (2008, p. 435–443), Plutchik (1980, p. 3–33), Circumplex theory of affect (Watson and Tellegen, 1985, p. 219–235)], EARL (Human-Machine Interaction Network on Emotion, HUMAINE), and WordNet–Affect (Sedding and Kazakov, 2004). According to Arribas-Ayllon et al. (2019, p. 619–648), Ekman's six basic emotions emerged as the most useful classification scheme for emotive language analysis in terms of ease of use by human annotators and training supervised machine learning algorithms. However, it has significant shortcomings, particularly in the representation of positive emotions. Plutchik (1980, p. 3–33)'s wheel of emotions provides a rich emotional spectrum but is complex and yields lower performance in machine learning. The Circumplex model (Watson and Tellegen, 1985, p. 219–235) offers a dimensional representation of emotions, capturing nuances well but also presenting complexity and moderate performance in machine learning. EARL (Human-Machine Interaction Network on Emotion, HUMAINE), designed for technological contexts, covers a wide range of emotions but has lower inter-annotator agreement and performance. Finally, WordNet–Affect (Sedding and Kazakov, 2004) is a rich lexical resource but is difficult to navigate and achieves the lowest agreement and performance.

In this study, selecting the appropriate scheme was challenging due to the extensive body of research and debate on these schemes. To find an appropriate scheme/classification of emotions to be used in this study, the following two main research objectives were set:

1. **The emotions scheme should be at least statistically and scientifically valid, and not only empirical and observational**.
2. The selected scheme should be derived from human annotators and supported by sufficient statistical validation. This ensures that the scheme is grounded in reliable human judgment and is statistically sound. Additionally, it must be acceptable to the community and widely used across various applications, ensuring its relevance and practical utility. Finally, the scheme should mitigate as many drawbacks of the existing schema as possible, addressing issues such as oversimplification and insufficient emotional coverage to provide a more comprehensive and nuanced understanding of emotions.
3. **The emotions scheme must be appropriate and aligned with the concepts of LDA**.
4. This alignment is necessary to ensure that the emotional classifications contribute meaningfully to the discovery of Lacanian discourses within the text. LDA examines language and its effects on the subject, emphasizing the importance of underlying structures and meanings in discourse. Therefore, the chosen scheme should capture these nuances and complexities in emotional expression, facilitating a deeper understanding of the text in line with Lacanian principles.

### 2.2 Related work

In recent years, quantitative discourse analysis in psychology has evolved into a robust interdisciplinary field, integrating methods from linguistics, computer science, and cognitive science. Researchers have increasingly turned to computational and statistical approaches to analyze how emotions are expressed and structured in discourse, using tools such as sentiment analysis, topic modeling, and latent semantic analysis (Tausczik and Pennebaker, 2009; Suleman and Korkontzelos, 2021; Lai et al., 2023).

Notably, research that focuses on language use as a psychological marker, with emotional language serving as a window into affective and mental states. For example, the Linguistic Inquiry and Word Count (LIWC) framework has been widely used to associate patterns of emotional word use with personality traits, well-being, and social dynamics (Pennebaker et al., 2003; Boyd and Pennebaker, 2017). These methods enable large-scale, systematic investigations of emotional discourse across diverse contexts – from social media and therapy sessions to autobiographical narratives (Kahn et al., 2007).

Furthermore, emotion classification in text using machine learning and NLP has enabled researchers to identify nuanced affective patterns and their correlations with psychological variables (Mohammad, 2021). Studies have examined how emotional valence, arousal, and discrete emotions such as anger and sadness are reflected in linguistic structures, providing quantitative insights into affective processes (Buechel and Hahn, 2017).

Although these approaches differ substantially from the structural and symbolic perspective of Lacanian psychoanalysis, they offer valuable frameworks for empirically grounding discourse-emotion relationships.

In this section, a few studies are briefly reviewed because of: (i) their background information, which helps to understand the context in which this study has been developed; and (ii) their ideas, which could be used to fuel future research.

In Bucci et al. (2022), the authors present two independent study cases.

The first is an Emotional Text Analysis (ETA). They show that emotions expressed in language are not individual phenomena but organizers of historically determined social relations. They conducted focus groups with young graduates who were about to start their first job in multinational corporations, as well as with their corporate mentors. Using ETA, they identified two distinct clusters of emotions. One was associated with young graduates' expectations, and the other with the mentors' accounts. In this case study, there is no mention of LDA.

The second case study is an example of LDA carried out within a therapeutic community (TC) for children and teenagers diagnosed with psychosis and autism spectrum disorders. Conversations between the therapeutic group and the family group were transcribed verbatim and reviewed by a panel of researchers trained in Lacanian psychoanalysis. The family group adopts a **Hysteric Discourse** and demands that the therapeutic group assume the **Master Discourse** and provide answers and solutions to the problem at hand. The leader of the therapeutic group wisely does not fall into the trap and adopts the **Analyst Discourse**, opening a path for further clarification of the problem.

This case study is closely related to this research and shows the possibility of applying LDA to daily dialogues and how the *Agent* changes roles as the dialog evolves.

In Vanheule (2016), the author provides a thorough revision of the four classic Lacanian Discourses that goes beyond the explanations given by J. Lacan himself at the time of his writings and by other researchers who repeated what J. Lacan said in simpler terms. For example, in the case of the **Master Discourse**, in addition to reinforcing that it is associated with authority and power, he includes:

In the **Master Discourse**, the insistent signifiers (**S1**) that provoke explanation (**S2**) come to the fore. Their articulation connotes the subject underlying this articulation of signifiers (**$**), and at the same time provokes an excitement (**a**) that is split off from the subject.

Next, the author delves into the **Capitalist Discourse**. The **Capitalist Discourse** is prevalent today and cannot be omitted from any study dealing with LDA and LDD. It underpins the rupture of social bonds and is the bedrock of many addictions that plague society. It has clinical importance because it must be managed differently depending on whether the patient is psychotic or neurotic.

In an important study focusing on the role of signifiers, the authors wrote in the Introduction of Olyff and Bazan (2023, p. 1):

Freud proposed that names of clinically salient objects or situations, for example, a beetle (*Käfer*) in Mr. E's panic attack, refer through their phonological form rather than their meaning, to etiologically important events—here, “*Que faire?*” which summarizes the indecisiveness of Mr. E's mother concerning her marriage with Mr. E's father. Lacan formalized these ideas, attributing full-fledged mental effectiveness to the signifier, and summarized this as “*the unconscious structured as a language*”. We tested one aspect of this theory, namely that there is an influence of the ambiguous phonological translation of the world upon our mental processing without us being aware of this influence.

The aforementioned work, published in 2023, is very recent and provides empirical evidence for the mental effectiveness of the signifier. It validates the empirical approach adopted by our own research and highlights the importance and possibility of identifying signifiers and the corresponding signifying chain that underpins LDA and LDD.

Robert “Rob” Haskell (1938–2010) served as a professor of psychology and department chair at the University of New England. His research areas include transfer of learning, small-group leadership, language and communication, **unconscious cognition** (Haskell, 2008), and analogical reasoning. He developed a novel **logico-mathematical, structural methodology for the analysis and validation of sub-literal (SubLit) language and cognition** (Haskell, 2003, p. 347–400).

His research, supported by several examples and a solid theoretical basis, demonstrates the presence of a hidden *Truth* within a discourse. The algorithm he proposed in Haskell (2003) has never been implemented and may be a valid dimension to include in future research to automatically identify signifiers and the associated signifying chain.

## 3 Adopted methodology

The adopted methodology comprises several decisions, which are discussed and justified in this section. They are:

- Choice of working only with texts.
- Choice of using dialogues.
- Choice of the emotions set.
- Emotions and discourses assignments, voting, and dataset creation process.
- Number of dialogues.
- Number of voters.
- Common-user's vote criteria for emotions and discourses.
- Probabilistic formulae to evaluate the relation between discourses and emotions.

### 3.1 Choice of the emotions set

In this section, solid arguments are presented for choosing a particular set of emotions that can be combined and used, in light of the discovery of the Five Lacanian Discourses, and for why they are aligned with the fundamental aspects of the LDA and LDD.

In the paper “Classifying Emotion: A Developmental Account,” (Zinck and Newen 2006) propose a systematic classification of emotions that accounts for their complexity and developmental stages. They distinguish between four developmental stages of emotions:

1. **Pre-emotions**: These are unfocused expressive emotional states, primarily observed in infants and characterized as either generally positive or negative.
2. **Basic emotions**: These emotions are innate and do not require cognitive processing. They include fear, anger, joy, and sadness.
3. **Primary cognitive emotions**: These emotions involve minimal cognitive content and are extensions or modifications of basic emotions.
4. **Secondary cognitive emotions**: These are highly complex emotions that*depend on cultural information and personal experience*. They developed within the four dimensions of the basic emotions and are enriched by cognitive mini-theories, resulting in more finely grained emotions.

Secondary cognitive emotions are particularly important because they depend on cultural information and personal experience, making them relevant for a nuanced analysis of emotional expression in texts. *Additionally, this category is correlated with the Lacanian Symbolic order, which must be considered when approaching a text within the field of LDA* (Frosh, 2013). This category also includes the emotions in the GoEmotions dataset, which was selected for this study.

The GoEmotions dataset, developed in Demszky et al. (2020), is the largest manually annotated dataset, comprising 58,009 English Reddit comments labeled for 27 emotions and Neutral. Created by researchers at Google Research, including Alan Cowen, a pioneer in emotion research, it offers a fine-grained typology adaptable to multiple downstream tasks, such as building empathetic chatbots or detecting harmful online behavior. The high quality of the annotations is demonstrated via Principal Preserved Component Analysis (PPCA), which shows reliable dissociation among the 27 emotion categories (Demszky et al., 2020).

Aligned with the classification proposed by Zinck and Newen (2006), the emotions in the GoEmotions dataset can be further categorized as follows:

- **Pre-Emotions**: Comfort.
- **Basic Emotions**: Sadness, Joy, Fear, and Anger.
- **Primary Cognitive Emotions**: Relief, Nervousness, Excitement, Disappointment, and Annoyance.
- **Secondary Cognitive Emotions**: Admiration, Approval, Caring, Confusion, Curiosity, Desire, Disapproval, Disgust, Embarrassment, Gratitude, Grief, Love, Optimism, Pride, Realization, Remorse, and Surprise.

The GoEmotions dataset is statistically valid, widely accepted, and suitable for a range of applications, including NLP. This extensive dataset is curated to provide a comprehensive and nuanced classification of emotions, aligning well with the developmental stages of emotions.

The robustness of the emotions in the dataset is demonstrated through various statistical analyses. For example, the dataset's annotations were found to be highly reliable, with 94% of examples having at least two raters agreeing on one label, and 31% having three or more raters in agreement. The high quality of these annotations is further validated by Principal Preserved Component Analysis (PPCA), which shows a strong dissociation among the 27 emotions. This ensures that the emotions captured in the dataset are both distinct and representative of a wide range of human emotional experiences.

Furthermore, the GoEmotions dataset has proven useful across various NLP applications. It provides a solid baseline for emotion classification models, particularly when fine-tuning models like BERT, yielding strong performance. This makes it well-suited for tasks such as building empathetic chatbots, analyzing customer feedback, and detecting harmful online behavior. The dataset's adaptability to multiple downstream tasks underscores its practical value and broad applicability.

In summary, the selection of this schema aligns perfectly with the two objectives stated in Section 2.1.5. First, the GoEmotions dataset meets the requirement that the schema be statistically and scientifically valid, not merely empirical and observational. The dataset's high reliability, demonstrated through rigorous statistical validation, ensures that it is grounded in sound human judgment and widely accepted in the community, fulfilling the first objective. Second, the selected schema is also appropriate and aligned with the concepts of LDA and LDD, as it captures the nuanced and complex emotional expressions necessary for meaningful discourse analysis in line with Lacanian principles. This ensures that this research is both methodologically sound and theoretically aligned, facilitating a deeper understanding of emotions within the framework of LDA and LDD.

To complete the set of emotions for this study, two additional emotions, “anguish” and “anxiety,” were added to the dataset because they are frequently cited in the psychoanalytic literature (see Sections 2.1.2, 2.1.1).

### 3.2 Choice of working only with texts

The current body of research lacks a comprehensive methodology for systematically identifying traces of Lacanian discourses across various modalities. While Parker (2010)'s study on LDA in interview texts provides valuable insights into identifying these discourses, it has not yet evolved into a fully developed framework for the systematic and efficient discovery of Lacanian discourses across different data types.

Due to the emerging nature of research in this field, this study focuses exclusively on textual data rather than incorporating modalities such as voice, sound, or video. Although relying solely on text may limit the exploration and identification of certain nuances inherent in Lacanian discourses, it offers a solid foundation for developing a methodological approach. Textual analysis provides a clear and structured starting point that can be refined and expanded in future research to include additional modalities. By beginning with text, the intent is to establish a robust analytical framework that can serve as a basis for more comprehensive studies in the future, introducing additional multimodalities as well.

### 3.3 Choice of using dialogues

As described in Section 2.1.3, Lacanian discourses consist of two integral components: the sender and the receiver of the discourse. To facilitate the exploration of potential emotions associated with these discourses, and given the limited prior research in this area, it is clear that textual dialogues are ideal for such examination. Dialogues reveal attributes and interpretive nuances that standalone or multi-paragraph texts do not provide.

Dialogues inherently reflect the foundational structure of Lacanian discourses, which involve communication between at least two parties conveying a message. As dialogues unfold, they reveal the latent information and intentions of the interacting parties. This dynamic flow of information makes it easier to identify the elements that constitute Lacanian discourses, i.e., the components **S1**, **S2**, **$**, and **a**.

Considering the aforementioned factors, the open-source dataset DAILY DIALOG (Li et al., 2017) has been selected, comprising everyday dialogues between two speakers. While other open-source datasets, such as Friends, could be employed, they have been written for specific purposes (e.g., screenplays intended for comedy or sarcasm) and might introduce bias and skew the study's results. Although these datasets may be useful in future research, as has been demonstrated by Joshi et al. (2016) and Poria et al. (2019), they do not align with the current research objectives.

The choice of this dataset was guided by the need for authentic, natural conversations that closely mirror real-life interactions. This authenticity is crucial for accurately analyzing the emotional and interpretive aspects of Lacanian discourses. Focusing on everyday dialogues aims to ensure that the discovered patterns and attributes are representative of genuine communication rather than artificially constructed scenarios.

### 3.4 Emotions and discourses assignments, voting and dataset creation process

To establish a theoretical correlation between Lacanian discourses and emotions, discourse and emotion annotations of dialogues were conducted using a custom-built platform. On this platform, each user was presented with a dialogue from the dataset and tasked with assigning discourses and emotions to each part of the dialogue. Along with these assignments, annotators provided the following metrics for each discourse or emotion:

**Confidence score of discourse:** This metric quantifies the level of certainty with which a discourse is assigned to a segment of the dialogue.

- **Scoring system:** The confidence score ranges from *Definitely Not, Probably Not, Probably Yes*, to *Definitely Yes*, representing varying levels of assurance.
- **Purpose:** This score helps gauge the reliability of the discourse assignment, ensuring that only strongly evidenced discourses receive higher confidence levels. By using this metric, a clear and reliable mapping between dialogues and Lacanian discourses can be established.

**Weight of discourse:** This value, ranging from 0 to 1, represents the strength or potency of the discourse within the dialogue. A higher weight indicates a stronger presence and influence of the discourse.

- **Significance:** A higher weight indicates a stronger presence and influence of the discourse. This metric provides insight into the significance of the discourse within the dialogue, assisting in prioritizing more influential discourses.
- **Application:** By evaluating the weight of each discourse, it is possible to identify the dominant discourses that shape the emotional and interpretative dynamics of the dialogue.

**Confidence score of emotion:** Similar to the confidence score of discourse, this metric measures the certainty of assigning a particular emotion to a segment of the dialogue.

- **Scoring system:** It uses the same *Definitely Not, Probably Not, Probably Yes*, to *Definitely Yes* scale to indicate the level of confidence in the emotional assignment.
- **Purpose:** This score ensures that emotional annotations are supported by strong evidence, enhancing the accuracy and reliability of the emotional analysis.

The voting process on the platform is designed to rigorously annotate dialogues with Lacanian discourses and emotions, supported by confidence and weight metrics. This method enables a systematic analysis of how discourses and emotions are intertwined, providing a foundation for understanding their theoretical correlations. By using a structured voting system, the annotations reflect both the presence and influence of discourses and emotions, contributing to a comprehensive exploration of Lacanian discourse theory in the context of emotional analysis. This annotation process lays the groundwork for statistical modeling and in-depth analysis to uncover potential correlations and patterns.

**Figure 1:**
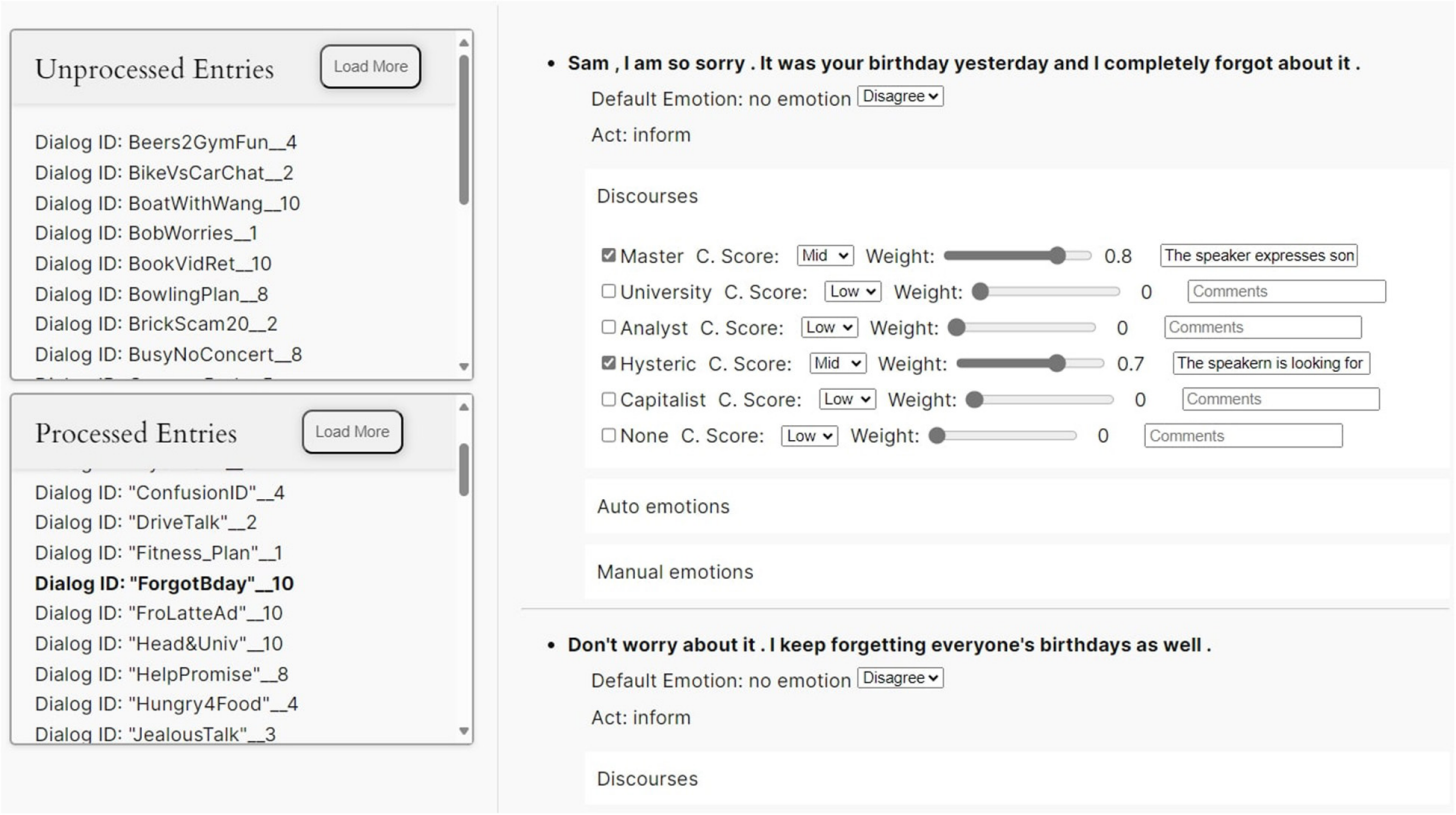
Screenshot of the platform used for the annotation process.

In Appendix, Figure 1 screenshot of the platform where voters annotated the dialogues is shown.

### 3.5 Probabilistic formulae to evaluate the relation between discourses and emotions

To derive a valid mathematical formula for evaluating the results of the annotation process regarding the relationship among discourses and emotions, the following methodology was adopted.

Let *S* be the set of all sentences annotated. The **conditional probability** of occurrence of a set of *n* discourses *d*_1_, *d*_2_, …, *d*_*n*_ (where 1 ≤ *n* ≤ 5) given a set of *l* emotions *e*_1_, *e*_2_, …, *e*_*l*_ (where 1 ≤ *l* ≤ 30) in a sentence of the dataset is defined by Equation 4:


$$\begin{array}{r} \text{Prob}\left(d_{1},d_{2},\ldots ,d_{n}|e_{1},e_{2},\ldots ,e_{l}\right)=\\ \frac{\sum_{s\in S_{d_{1},\ldots ,d_{n},e_{1},\ldots ,e_{l}}}\left(\prod_{i=1}^{n}c_{s}\left(d_{i}\right)\cdot \prod_{j=1}^{l}c_{s}\left(e_{j}\right)\right)}{\sum_{s\in S_{e_{1},\ldots ,e_{l}}}\left(\prod_{i=1}^{k_{s}}c_{s}\left(d_{i}\right)\cdot \prod_{j=1}^{l}c_{s}\left(e_{j}\right)\right)} \end{array}$$ (4)

where:

- *c*_*s*_(*d*_*i*_) is the **confidence score** assigned to the occurrence of the discourse **d_*i*_**
- *c*_*s*_(*e*_*j*_) is the **confidence score** assigned to the occurrence of the emotion **e_*j*_** in sentence *s* (where *s*∈*S*), for *j* = 1, 2, …, *l*. Here, *e*_*j*_ (where 1 ≤ *j* ≤ 30) represents one of the 30 emotions.

The numerator in the formula is a summation over a subset *S*_*d*_1_, …, *d*_*n*_, *e*_1_, …, *e*_*l*__, which includes all sentences *s* ∈ *S* that contain the discourses *d*_1_, …, *d*_*n*_ and the emotions *e*_1_, …, *e*_*l*_. Within this sum, the product of the confidence scores *c*_*s*_(*d*_*i*_) for each discourse *d*_*i*_ and the confidence scores *c*_*s*_(*e*_*j*_) for each emotion *e*_*j*_ is included. This represents the aggregated confidence for instances where the discourses *d*_1_, *d*_2_, …, *d*_*n*_ and the emotions *e*_1_, *e*_2_, …, *e*_*l*_ co-occur within the dataset.

The denominator sums over a subset *S*_*e*_1_, …, *e*_*l*__, which includes all sentences *s*∈*S* that contain the emotions *e*_1_, …, *e*_*l*_, without any requirement on co-occurring discourses in the sentences. The denominator involves a product of confidence scores, but here the set of discourses *d*_*i*_ may vary, as indicated by the product $\prod_{i=1}^{k_{s}}$; where *k*_*s*_ is the number of discourses in the sentence *s*. This part of the formula represents the total aggregated confidence for all occurrences of the emotions *e*_1_, *e*_2_, …, *e*_*l*_, regardless of the number of discourses they appear with, i.e, not only when the examined discourses *d*_1_…*d*_*n*_ occur. The denominator, therefore, captures the overall likelihood of the emotions occurring within the dataset, independently of the specific discourses associated with them.

**Theorem 3.1** (The probability estimator defined in Equation 4 is unbiased.). *Proof. We exemplify in the case of* Pr{*d*_1_∣*e*_1_}; *the proof can be easily extended to all possible combinations of discourses and emotions*.

*Let *p* be the true, unknown probability*


$$p=\mathrm{Pr}\left\{d_{1}|e_{1}\right\}$$

*of occurrence of (only) discourse d*_1_ *exclusively with emotion e*_1_. *According to Equation 4, our probability estimator for p is*:


$$\begin{array}{l} \hat{p}=\frac{\underset{s\in S_{d_{1},e_{1}}}{\sum }c_{s}\left(d_{1}\right)\cdot c_{s}\left(e_{1}\right)}{\underset{s\in S_{e_{1}}}{\sum }\left(\overset{k_{s}}{\underset{i=1}{\prod }}c_{s}\left(d_{i}\right)\right)\cdot c_{s}\left(e_{1}\right)}=\frac{A}{\text{Π}}\; \end{array}$$ (5)

*where the numerator A sums up the total confidence on the exclusive co-occurrence of discourse d*_1_ *with emotion e*_1_, *while the denominator Π sums up the total confidence on the occurrence of emotion e*_1_; *since we condition on the occurrence of emotion e*_1_, *then Π can be considered constant (the “new” probability space created by conditioning), and the conditional probability $\hat{p}$ depends on the fraction of sentences when e*_1_ *exclusively co-occurs with d*_1_.

*We note that when* (*d*_1_, *e*_1_) *co-occur exclusively, then*


$$\begin{array}{l} {\text{c}}_{s}\left(d_{1}\right)\cdot c_{s}\left(e_{1}\right)=\left(\overset{k_{s}}{\underset{i=1}{\prod }}c_{s}\left(d_{i}\right)\right)\cdot c_{s}\left(e_{1}\right)\; \end{array}$$ (6)

*because in such a case the right-hand side of Equation 6 clearly becomes equal to the left-hand side.*

*Such exclusive co-occurrence of* (*d*_1_, *e*_1_) *happens with probability Pr*{*d*_1_|*e*_1_} = *p*. *Let N* = |*S*_*e*_1__| *the number of sentences where emotion e*_1_ *occurs and let i* (1 ≤ *i* ≤ *N*) *a given sentence in S*_*e*_1__. *Then the confidence on the desired d*_1_, *e*_1_ *exclusive co-occurrence is*:


$$C_{i}=\{\begin{array}{ll} c_{s}\left(d_{1}\right)\cdot c_{s}\left(e_{1}\right) & ,\text{with probability }p\\ 0 & ,\text{otherwise} \end{array}$$

*so*


$$\begin{array}{l} 𝔼\left(C_{i}\right)=c_{s}\left(d_{1}\right)\cdot c_{s}\left(e_{1}\right)\cdot p \end{array}$$ (7)

*where* 𝔼[·] *symbolizes expectation*.

*The total confidence in d*_1_, *e*_1_ *exclusive co-occurrence across all sentences is*:


$$C=\overset{N}{\underset{i=1}{\sum }}C_{i}$$

*so, by the linearity of expectation property, it is*:


$$𝔼\left(C\right)=𝔼\left(\overset{N}{\underset{i=1}{\sum }}C_{i}\right)=\overset{N}{\underset{i=1}{\sum }}𝔼\left(C_{i}\right)\Rightarrow$$

$$𝔼\left(A\right)=𝔼\left(\underset{s\in S_{d_{1},e_{1}}}{\sum }c_{s}\left(d_{1}\right)\cdot c_{s}\left(e_{1}\right)\cdot p\right)\text{ because of Equations 5, 7}$$

$$=p\cdot 𝔼\left[\left(\overset{k_{s}}{\underset{i=1}{\prod }}c_{s}\left(d_{i}\right)\right)\cdot c_{s}\left(e_{1}\right)\right]\text{ due to Equation 6}$$

=p·𝔼(Π)=p·Π

*because, as explained above*, Π *is constant. Thus*


$$𝔼\left(\hat{p}\right)=𝔼\left(\frac{A}{\text{Π}}\right)=\frac{1}{\text{Π}}𝔼\left(A\right)=\frac{1}{\text{Π}}p\cdot \text{Π}=p$$

*so the estimator* $\hat{p}$ *is unbiased*.

This general formula can be adapted to calculate the probability for any number of discourses n (1 ≤ *n* ≤ 5) given any number of emotions l (1 ≤ *l* ≤ 30). By extending the summation and product operations accordingly, the method remains applicable whether one is evaluating the appearance of a single discourse, a set of multiple discourses, or a combination of several emotions. The framework is flexible, allowing for the inclusion of more complex discourse-emotion relationships within the dataset, ensuring comprehensive analysis across different scenarios.

For example, the general formula of Equation 4 can be applied to any specific case involving different combinations of discourses and emotions, as shown below:

- **Example 1: One discourse, one emotion:**
  $$\text{Prob}\left(d_{1}|e_{1}\right)=\frac{\sum_{s\in S_{d_{1},e_{1}}}c_{s}\left(d_{1}\right)\cdot c_{s}\left(e_{1}\right)}{\sum_{s\in S_{e_{1}}}\left(\prod_{i=1}^{k_{s}}c_{s}\left(d_{i}\right)\right)\cdot c_{s}\left(e_{1}\right)}$$
  Here, the numerator sums the product of the confidence scores for discourse *d*_1_ given emotion *e*_1_ across all sentences. The denominator sums the product of the confidence scores for all occurrences of emotion *e*_1_ with various discourses.
- **Example 2: Two discourses, one emotion:**
  $$\text{Prob}\left(d_{1},d_{2}|e_{1}\right)=\frac{\sum_{s\in S_{d_{1}d_{2},e_{1}}}c_{s}\left(d_{1}\right)\cdot c\left(d_{2}\right)\cdot c_{s}\left(e_{1}\right)}{\sum_{s\in S_{e_{1}}}\left(\prod_{i=1}^{k_{s}}c_{s}\left(d_{i}\right)\right)\cdot c_{s}\left(e_{1}\right)}$$
  Here, the numerator sums the product of the confidence scores for discourses *d*_1_ and *d*_2_ given emotion *e*_1_ across all sentences. The denominator sums the product of the confidence scores for all occurrences of emotion *e*_1_ with the discourses *d*_1_ and *d*_2_.
- **Example 3: Two discourses, two emotions:**
  $$\text{Prob}\left(d_{1},d_{2}|e_{1},e_{2}\right)=\frac{\sum_{s\in S_{d_{1}d_{2},e_{1}e_{2}}}c_{s}\left(d_{1}\right)\cdot c_{s}\left(d_{2}\right)\cdot c_{s}\left(e_{1}\right)\cdot c_{s}\left(e_{2}\right)}{\sum_{s\in S_{e_{1}e_{2}}}\left(\prod_{i=1}^{k_{s}}c_{s}\left(d_{i}\right)\right)\cdot c_{s}\left(e_{1}\right)\cdot c_{s}\left(e_{2}\right)}$$
  In this case, the numerator sums the product of the confidence scores for discourses *d*_1_ and *d*_2_ given emotions *e*_1_ and *e*_2_ across all sentences. The denominator sums the product of the confidence scores for all occurrences of the emotions *e*_1_ and *e*_2_ with the discourses *d*_1_ and *d*_2_.

A justification for the above complex probability formulation is needed. Indeed, a simpler formulation could have been adopted, where the probability would be the fraction of co-occurrences of the examined discourse-emotion combinations over the occurrences of the considered emotions. However, such an approach could overlook crucial factors, including annotation confidence for discourse, discourse strength, and confidence in the associated emotions. An additional important factor that needs to be addressed is the “discourse weight” *w*_*di*_, which captures the “strength” of the occurrence of the discourse in a sentence, i.e., how prominently the discourse occurs in the sentence. The inclusion of these three factors provides additional insight into the co-occurrence of the discourse with the emotions.

Table 1 presents the confidence scores for discourse *d*_1_, the weight of discourse *d*_1_, and the confidence scores for *e*_1_ in a hypothetical dataset, where *d*_1_ and *e*_1_ appear together in only four sentences.

**Table 1:** An example showing the confidence scores for discourse *d*_1_, the weight of discourse *d*_1_, and the confidence scores for *e*_1_ in a hypothetical dataset, where *d*_1_ and *e*_1_ appear together in only four sentences.

**Sentence**	**Conf (d1)**	**Conf (e1)**	**Weight (d1)**
Sentence 1	0.2	0.4	1
Sentence 2	0.3	0.2	0.8
Sentence 3	0.3	0.3	0.7
Sentence 4	0.2	0.2	0.4

For the data in Table 1, even when confidence levels are low, the probability remains high due to the limited annotated diversity in the small dataset, leading to an almost exclusive correlation. Specifically, as *e*_1_ only appears with *d*_1_ the conditional probability Prob(*d*_1_|*e*_1_) is as follows:


$$\begin{array}{r} \text{Prob}\left(d_{1}|e_{1}\right)=\\ \frac{\left(0.2\times 0.4\right)+\left(0.3\times 0.2\right)+\left(0.3\times 0.3\right)+\left(0.2\times 0.2\right)}{\left(0.2\times 0.4\right)+\left(0.3\times 0.2\right)+\left(0.3\times 0.3\right)+\left(0.2\times 0.2\right)}=1 \end{array}$$

In other words, even this sophisticated probability definition falls short of adequately modeling the relationship between emotions and discourses. Therefore, it is necessary to consider the strength of the discourse's presence. This is achieved by incorporating the weights of the discourses as described in Definition 3.1.

**Definition 3.1** (Weight level). *The **weight level (W)** of the co-occurrence of the discourses d*_1_, *d*_2_, …, *d*_*n*_ *with emotions e*_1_, *e*_2_, …, *e*_*l*_ *is defined as follows*:

*(i) First, for each sentence s where this combination of discourses and emotions occurs, the product of the involved discourse weights w*_*s*_(*d*_*i*_) (*where* 1 ≤ *i* ≤ *n*) *is taken*.
*(ii) Then, to evaluate the total discourse weight level, the above product is summed over all relevant sentences s where the examined combination occurs*.

*The weight level is then given by*:


$$\text{W}\left(d_{1},d_{2},\ldots ,d_{n},e_{1},e_{2},\ldots ,e_{l}\right)=\underset{s\in S_{d_{1}\ldots d_{n},e_{1},\ldots ,e_{l}}}{\sum }\overset{n}{\underset{i=1}{\prod }}w\left(d_{i}\right)$$

□

The relation R among discourses *d*_1_, *d*_2_, …*d*_*n*_ and emotions *e*_1_, *e*_2_, …, *e*_*l*_ is given in Definition 3.2.

**Definition 3.2** (Relation of co-occurrence). *The **relation** of the co-occurrence of the discourses d*_1_, *d*_2_, …, *d*_*n*_ *with emotions e*_1_, *e*_2_, …, *e*_*l*_ *is defined by Equation 8, as follows*:


$$\begin{array}{r} R\left(d_{1},d_{2},\ldots ,d_{n},e_{1},e_{2},\ldots ,e_{l}\right)=\\ \text{Prob}\left(d_{1},d_{2},\ldots ,d_{n}|e_{1},e_{2},\ldots ,e_{l}\right)\times W\left(d_{1},d_{2},\ldots ,d_{n},e_{1},e_{2},\ldots ,e_{l}\right) \end{array}$$ (8)

□

Using the data provided in Table 1, the weight level and the relation between *d*_1_ and *e*_1_ are calculated as


$$W\left(d_{1}|e_{1}\right)=1\times 0.8\times 0.7\times 0.4=0.224$$

and


$$R\left(d_{1}|e_{1}\right)=Pr\left(d_{1}|e_{1}\right)\times W\left(d_{1}|e_{1}\right)=1\times 0.224=0.224$$

Interestingly, the discourse-emotion relationship now admits fine-grained values, possibly covering a very broad spectrum regardless of the dataset size, because all factors of the discourse-emotion relationship are now directly taken into account.

This result is then normalized, ensuring that the final value lies within a standard range of 0 to 1 where the maximum value is taken from all relations where the number of discourses is *n* and the number of emotions *l*. This final relation is named the **relation intensity (RI)** and described by Definition 3.3, for the co-occurrence of a certain combination of discourses *d*_1_, *d*_2_, …, *d*_*n*_ with certain emotions *e*_1_, *e*_2_, …, *e*_*l*_ and given by Equation 9:

**Definition 3.3** (Normalized relation intensity). *The **normalized relation intensity** among discourses d*_1_, *d*_2_, …, *d*_*n*_ *and emotions e*_1_, *e*_2_, …, *e*_*l*_ *is defined as follows*:


$$\begin{array}{r} \mathrm{RI}\left(d_{1},d_{2},\ldots ,d_{n},e_{1},e_{2},\ldots ,e_{l}\right)=\\ \frac{R\left(d_{1},d_{2},\ldots ,d_{n},e_{1},e_{2},\ldots ,e_{l}\right)}{\max_{n,l}\left(R\left(d_{i1},d_{i2},\ldots ,d_{in},e_{j1},e_{j2},\ldots ,e_{jl}\right)\right)} \end{array}$$ (9)

## 4 Results and discussion

In this section, the experimental procedure, the processing of assignments (from now on designated as votes), and the results and their discussion are detailed.

### 4.1 Experimental procedure

The experimental procedure is carried out entirely on the custom-built platform. The platform ensures that voters are aware of the meaning and purpose of the discourses' confidence score and weight, as well as the emotions' confidence score. Furthermore, the platform requires that emotions be selected from the chosen dataset and that voting be possible in combinations of emotions and discourses.

- 40 dialogues were randomly selected from the database corresponding to 264 sentences.
- Three voters, familiar with the Lacanian Theory of Discourses, independently from each other, for each sentence:
  – elected emotions from the pre-defined set, and assigned a confidence score. The voters were instructed to elect up to three emotions, i.e., they were not restricted to choose just one emotion;
  – elected the appropriate discourses and assigned both a confidence score and a weight as described in Section 3.4.
- The assignments of the three voters were processed and combined into the votes of a so-called “common user” for both the assigned discourses and emotions as well.

The emotions and discourses assignment is essentially a subjective process. As such, it leads to disagreements and discrepancies in assignments that we prefer to label as diversity. In some cases, diversity simply means a disagreement that does not contribute to identifying any pattern. In other cases, diversity is a desirable feature of the process because it may be informative and enrich the perception of what is hidden behind the manifest narratives.

To quantitatively evaluate inter-rater agreement, we calculated agreement scores for all combinations of raters across the five Lacanian discourses using Krippendorff (2010)'s alpha coefficient. Krippendorff's alpha coefficient is a statistical measure used to quantify the agreement among multiple observers when coding or rating a set of items. When considering all three raters, the agreement scores were 0.70 for the Analyst discourse, 0.60 for the University discourse, 0.55 for both the Master and Capitalist discourses, and 0.52 for the Hysteric discourse. Examining pairwise agreement revealed more variability. The agreement between Voter 1 and Voter 2 was generally higher, reaching substantial levels for the Analyst (0.80) and University (0.71) discourses. Similarly, Voter 1 and Voter 3 showed strong agreement, particularly for the Capitalist (0.89), Analyst (0.72), and Master (0.67) discourses. The agreement between Voter 2 and Voter 3 was more moderate, with the highest score being 0.58 for the Analyst discourse.

The pseudocode for the algorithm to derive the final discourses for the sentences is Appendix Algorithm 2, and its simplified version is shown in Table 2.

**Table 2:** Summary of rules to derive the discourses assignment of the “common user.”

**Rules**	**Voter 1**	**Voter 2**	**Voter 3**	**Outcome (discourse, confidence score, and weight)**
Rule 1	*d*_1_	*d*_1_	*d*_1_	(*d*_1_, H, 1)
Rule 2	*d*_1_ *d*_2_	*d*_1_ *d*_2_	*d*_1_ *d*_2_	(*d*_1_, H, 1) (*d*_2_, H, 1)
Rule 3	*d*_1_ *d*_2_	*d*_1_ *d*_2_	*d*_1_	(*d*_1_, H, 1) (*d*_2_, M, 1)
Rule 4	*d*_1_ *d*_2_	*d*_1_	*d*_1_	(*d*_1_, H, 0.8)
Rule 5	*d*_1_ *d*_2_ *d*_3_	*d*_1_	*d*_1_	(*d*_1_, H, 0.6)
Rule 6	*d*_1_ *d*_2_	*d*_1_ *d*_3_	*d*_1_	(*d*_1_, H, 0.6)
Rule 7	*d*_1_ *d*_2_ *d*_3_	*d*_1_ *d*_2_	*d*_1_ *d*_2_	(*d*_1_, H, 0.8) (*d*_2_, H, 0.8)
Rule 8	*d*_1_	*d*_1_	(none)	(*d*_1_, M, 1)
Rule 9	*d*_1_	*d*_1_	*d*_2_	(*d*_1_, M, 1)
Rule 10	*d*_1_	*d*_1_ *d*_3_	*d*_2_	(*d*_1_, M, 0.8)
Rule 11	*d*_1_ *d*_2_	*d*_2_ *d*_3_	*d*_1_ *d*_2_	(*d*_1_, M, 1) (*d*_2_, H, 0.8)
Rule 12	*d*_1_ *d*_2_	*d*_1_	*d*_2_	(*d*_1_, M, 1) (*d*_2_, M, 1)
Rule 13	*d*_1_ *d*_2_	*d*_1_ *d*_3_	(none)	(*d*_1_, M, 0.6)
Rule 14	*d*_1_ *d*_2_	*d*_1_ *d*_2_	(none)	(*d*_1_, M, 1) (*d*_2_, M, 1)
Rule 15	*d*_1_ *d*_2_	*d*_1_ *d*_2_	*d*_3_ *d*_4_	(*d*_1_, M, 1) (*d*_2_, M, 1)
Rule 16	*d*_1_	*d*_1_	*d*_3_ *d*_4_	(*d*_1_, M, 1)
Rule 17	*d*_1_	*d*_1_ *d*_2_	*d*_2_	(*d*_1_, M, 1) (*d*_2_, M, 1)
Rule 18	*d*_1_ *d*_4_	*d*_2_ *d*_3_ *d*_4_	*d*_1_ *d*_2_ *d*_3_	(*d*_1_, M, 1) (*d*_2_, M, 1) (*d*_3_, M, 1) (*d*_4_, M, 1)
Rule 19	*d*_1_	*d*_1_ *d*_2_	*d*_2_ *d*_3_	(*d*_1_, M, 1) (*d*_2_, M, 0.8)
Rule 20	*d*_1_	*d*_2_ *d*_3_ *d*_4_	*d*_2_	(*d*_2_, M, 0.6)
Rule 21	(none)	*d*_1_ *d*_2_	(none)	(none, L, 0)
Rule 22	*d*_1_ *d*_2_	*d*_1_ *d*_2_	*d*_1_ *d*_3_	(*d*_1_, H, 0.8) (*d*_2_, M, 1)
Rule 23	*d*_1_	(none)	(none)	(none, L, 0)
Rule 24	*d*_1_ *d*_2_	*d*_1_	*d*_3_	(*d*_1_, M, 0.8)
Rule 25	*d*_1_ *d*_2_	*d*_1_ *d*_2_	*d*_3_	(*d*_1_, M, 1) (*d*_2_, M, 1)
Rule 26	*d*_1_	*d*_1_ *d*_2_	*d*_1_ *d*_2_ *d*_3_	(*d*_1_, H, 0.8) (*d*_2_, M, 0.8)
Rule 27	*d*_1_ *d*_2_	*d*_1_	(none)	(*d*_1_, M, 0.8)
Rule 28	*d*_1_ *d*_2_	*d*_1_ *d*_3_	*d*_2_ *d*_3_	(*d*_1_, M, 1) (*d*_2_, M, 1) (*d*_3_, M, 1)
Rule 29	*d*_1_ *d*_2_	*d*_1_ *d*_3_	*d*_2_	(*d*_1_, M, 0.8) (*d*_2_, M, 1)

Let's take, for example, the algorithm's Rule 11:

- Voter 1: *d*_1_ *d*_2_;
- Voter 2: *d*_2_ *d*_3_;
- Voter 3: *d*_1_ *d*_2_;
- Outcome
  – (*d*_1_, *M*, 1): is kept because it appears in 2 out of 3 votes; its confidence score is M(edium) because it was not selected by Voter 2; its weight is 1 because it does not appear together with a discourse that has been discarded.
  – (*d*_2_, *H*, 0.8): is kept because it appears in 3 out of 3 votes; its confidence score is H(igh) because it was selected by all voters; its weight is 0.8 because it appears along *d*_3_ that was discarded.
  – *d*_3_: discarded because it was selected by only 1 voter.

In this case, *d*_3_ is a discrepancy that does contribute to the understanding of the enunciation.

Let us take, for example, the algorithm's Rule 18:

- Voter 1: *d*_1_ *d*_4_;
- Voter 2: *d*_2_ *d*_3_ *d*_4_;
- Voter 3: *d*_1_ *d*_2_ *d*_3_;
- Outcome
  – (*d*_1_, *M*, 1): is kept because it appears in 2 out of 3 votes; its confidence score is M(edium) because it was not selected by Voter 2; its weight is 1 because it does not appear together with a discourse that has been discarded.
  – (*d*_2_, *M*, 1): is kept because it appears in 2 out of 3 votes; its confidence score is M(edium) because it was not selected by Voter 1; its weight is 1 because it does not appear together with a discourse that has been discarded.
  – (*d*_3_, *M*, 1): is kept because it appears in 2 out of 3 votes; its confidence score is M(edium) because it was not selected by Voter 1; its weight is 1 because it does not appear together with a discourse that has been discarded.
  – (*d*_4_, *M*, 1): is kept because it appears in 2 out of 3 votes; its confidence score is M(edium) because it was not selected by Voter 3; its weight is 1 because it does not appear together with a discourse that has been discarded.

If the criterion were to keep only the discourses that achieved consensus, none of the discourses would have been selected. However, in this case, any discourse is selected by more than 1 voter and is informative, which makes the combination of discourses (*d*_1_, *d*_2_, *d*_3_, *d*_4_) to be taken into account.

Let us take, for example, the algorithm's Rule 22:

- Voter 1: *d*_1_ *d*_2_;
- Voter 2: *d*_1_ *d*_2_;
- Voter 3: *d*_1_ *d*_3_;
- Outcome
  – (*d*_1_, *H*, 0.8): is kept because it appears in 3 out of 3 votes; its confidence score is H(igh) because it was selected by all voters; its weight is 0.8 because it appears along *d*_3_ that was discarded.
  – (*d*_2_, *M*, 1): is kept because it appears in 2 out of 3 votes; its confidence score is M(edium) because it was not selected by Voter 3; its weight is 1 because it does not appear together with a discourse that has been discarded.
  – *d*_3_: discarded because it was selected by only 1 voter.

In this case, *d*_3_ is a discrepancy that does contribute to the understanding of the enunciation. If the criterion were to keep only the discourses that achieved consensus, *d*_2_ would have been discarded. However, *d*_2_ is informative and makes the combination of discourses (*d*_1_, *d*_2_) to be taken into account.

It may seem that the weights assigned by individual voters are not important because they are not considered when deriving the weight assigned by the “common user.” The weights assigned by individual voters help voters reflect their perceptions. In future research, a comparison will be made between the relationships obtained by individual voters, and in this case, these weights are essential.

The algorithm for deriving the discourses for the “common user's” vote considers only up to 4 discourses, as this was the maximum number a voter assigned to a sentence. Also, the cases that led to a discourse of “none” were discarded from the final analysis of the data.

Hereafter, an example of dialog processing is shown to help with understanding the “common user's” vote evaluation.

*Dialog ID: FroLatteAd*_10

**I can't believe Mr. Fro didn't buy it. Who does that guy think he is anyway? Bill Gates?**

- Voter 1: Hysteric, High, 0.9;
- Voter 2: Hysteric, Mid, 0.7;
- Voter 3: Hysteric, High, 1.0;
- Common user—Rule 1: Hysteric, High, 1.0.

**He had a lot of nerve telling us our ads sucked**.

- Voter 1: University, Mid, 0.6; Hysteric, Low, 0.2;
- Voter 2: Master, Low, 0.1; Hysteric, Mid, 0.6;
- Voter 3: Hysteric, High, 1.0;
- Common user—Rule 6: Hysteric, High, 0.6.

**Time to order. Balista, today I want a skinny triple latte**.

- Voter 1: Hysteric, Mid, 0.4;
- Voter 2: Hysteric, Low, None; Capitalist, Low, 0.5;
- Voter 3: Hysteric, High, 0.8;
- Common user—Rule 4: Hysteric, High, 0.8.

**When did you start worrying about your weight?**

- Voter 1: Analyst, High, 0.8;
- Voter 2: Analyst, Mid, 0.7;
- Voter 3: Analyst, High, 0.5; Hysteric, High, 0.5.
- Common user—Rule 4: Analyst, High, 0.8.

**I'm not. I just don't feel like drinking whole milk today. Why? Do you think I'm fat?**

- Voter 1: Master, Mid, 0.5; Hysteric, High, 0.8;
- Voter 2: Master, Low, 0.2; Hysteric, Mid, 0.7;
- Voter 3: Master, High, 0.5; Hysteric, High, 0.5;
- Common user—Rule 2: Master, High, 1.0; Hysteric, High, 1.0.

**No, Jess, chill out!**

- Voter 1: Master, Low, 0.2;
- Voter 2: Master, Low, 0.6; Hysteric, Low, 0.1;
- Voter 3: Master, High, 0.8;
- Common user—Rule 4: Master, High, 0.8.

The example shows that:

- The algorithm harmonizes the discrepancies among the individual voters.
- The harmonization is accomplished by discarding votes that are given just by one voter, and decreasing the value of the confidence score and the weight level in cases where the votes are not unanimous.
- The confidence score and weight level assigned by the individual voters are not taken into account in the derivation of the “common user's” vote. Nevertheless, these individual assignments are important when deriving the model for the individual voters. However, such modeling is out of the scope of the current research.
- The algorithm enables the capture of the presence of both unique discourses and traces of multiple discourses in each of the statements.

The algorithm to derive the emotions for the “common user's” vote is shown in the Algorithm 2.

### 4.2 Votes processing

Equation 4 is applied to the “common user's” votes, and a complete table of all conditional probabilities is generated. Table 3 shows an extract from the complete table of conditional probabilities. In any given row, the first column is a set of up to three simultaneous emotions, and the remaining columns show the conditional probabilities for the set of discourses. It may be seen that the sum of the probabilities in each row is 1. In the complete table, the sum of the probabilities in each column is also 1.

**Table 3:** Conditional probabilities for emotional sets (M, Master; U, University; A, Analyst; H, Hysteric; C, Capitalist).

**Emotion**	**A, C**	**A, H**	**A, H, M**	**A, M**	**C, H**	**H, M**	**H, M, U**		**M, U**	**A**	**C**	**H**	**M**	**U**
(“Annoyance,” “disappointment,” “disapproval”)	0	0.0884	0	0	0	0.6389	0.075953		0	0	0	0.1136	0.0830	0
(“Admiration,” “approval,” “excitement”)	0	0	0	0	0	0	0.049467		0	0	0	0.8617	0	0
(“Anger,” “annoyance,” “disapproval”)	0	0	0	0	0	0.0491	0		0	0	0	0.7052	0.1146	0.1310
(“Admiration,” “approval,” “desire”)	0.1926	0	0	0	0	0	0		0	0	0	0.8074	0	0
(“Annoyance,” “disapproval,” “realization”)	0	0	0	0	0	0	0.5453		0	0	0	0.4547	0	0

Next, Equation 9 is applied to the conditional probabilities, generating a complete table of the relation intensity between a set of emotions and a set of discourses. In any given row, the first column lists up to three simultaneous emotions, and the remaining columns show the relation intensity for all 13 discourse combinations considered.

For clarity, we symbolized the relation intensity for particular combinations of discourses with emotions


$$\text{R}\text{I}\left(d_{1},d_{2},\ldots ,d_{n},e_{1},e_{2},\ldots ,e_{l}\right)$$

in the following way:


$$\left\{e_{1},e_{2},\ldots ,e_{l}\right\}\text{R}\text{I}\left\{d_{1},d_{2},\ldots ,d_{n}\right\},$$

making it easier the identification of the involved emotions and discourses.

In general terms, this relation intensity can be represented as:


$$\left\{e_{i},e_{j},e_{k}\right\}\text{R}\text{I}\left\{d_{a},d_{b},d_{c}\right\}=r_{m},$$

where 0 ≤ {*i, j, k*} ≤ 30. The value 0 is used to represent “no emotion considered” and the other values point to a specific emotion of the dataset.

{*a, b, c*} represent one of the five Lacanian discourses or none of them to take into account a single or a combination of discourses.

1 ≤ *m* ≤ 13 represents one of the 13 possible discourses combinations and 0 ≤ *r*_*m*_ ≤ 1. As already shown by Equation 9, *r*_*m*_ is obtained by the multiplication of a probability and a weight, both of which are less or equal to 1, and a further normalization. So, it is not possible to have a high value of the relation intensity if the corresponding conditional probability is low. For each set of emotions {*e*_*i*_, *e*_*j*_, *e*_*k*_} the 13 values of *r*_*m*_ can be sorted in descending order, indicating the most prevalent discourses associated with that set of emotions.

**Figure 2:**
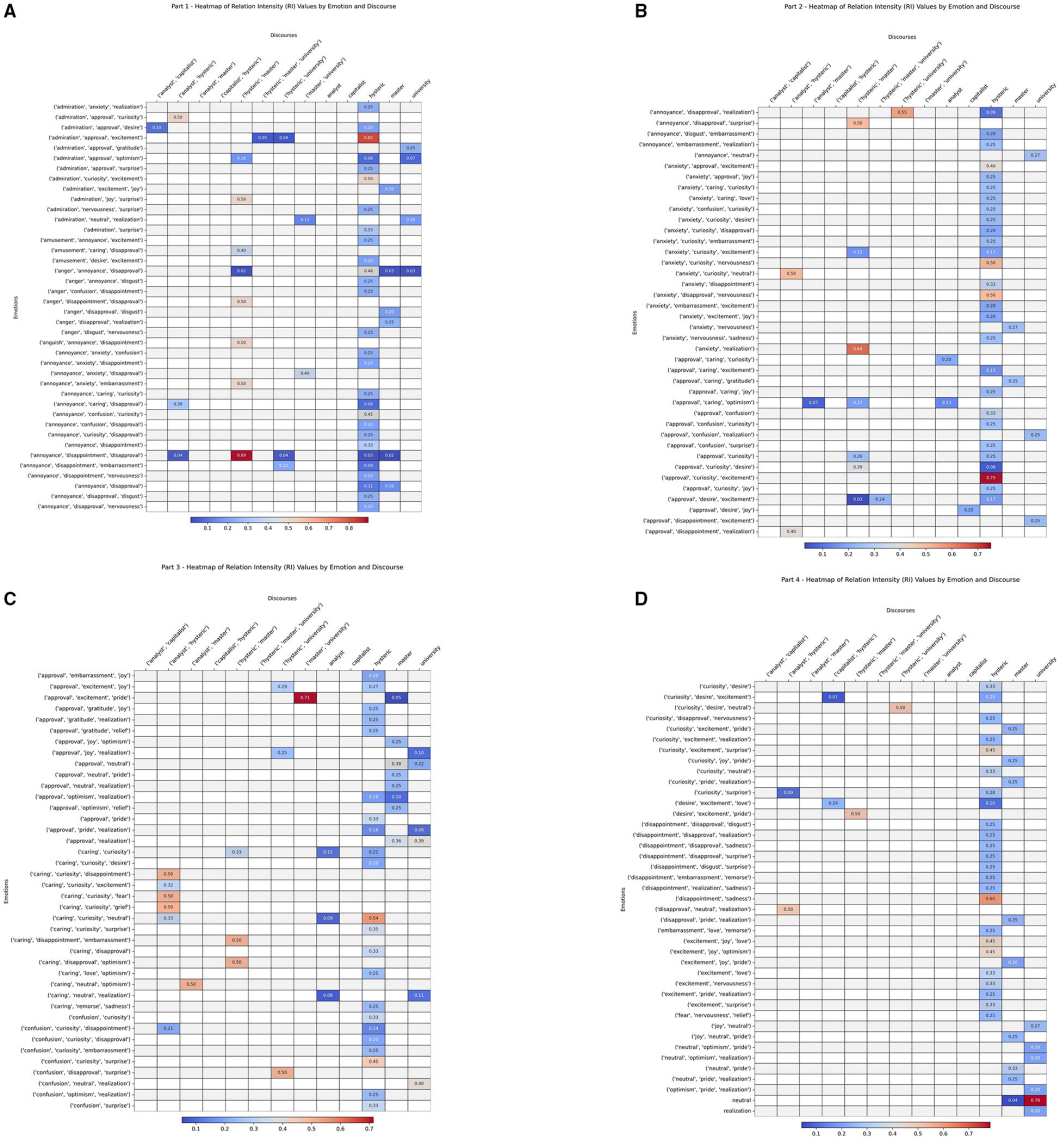
The complete heat map of the relation intensity between emotions and Lacanian discourses displayed in a 2x2 grid. Each subfigure represents a specific section of the heatmap: **(A)** Part 1 (top-left), **(B)** Part 2 (top-right), **(C)** Part 3 (bottom-left), and **(D)** Part 4 (bottom-right).

The relation intensity results have been summarized in a heat map shown in Figure 2, presented as a 2 × 2 grid of subfigures. Each subfigure highlights one quadrant of the heat map, focusing on the top 5 relation intensity values for each combination of emotions. While the complete relation intensity table has 198 rows, the table limited to the top 5 values includes only 67 rows.

These heat maps show the entire dataset and are not limited to just the top 5.

### 4.3 Findings and discussion

It is important to point out that the findings and corresponding explanations are preliminary due to the limitations of the experimental procedure and decisions taken for the adopted methodology. Nevertheless we are confident that the qualitative findings are of significance and the quantitative ones deserve to be refined in future work.

Before showing the results, it is necessary to clarify the relevance and consequences of working with a combination of emotions instead of a single emotion for each sentence.

We used the term sentence to refer to a complete enunciation by a speaker in each part of the dialogue. We did not break each enunciation into its constituent parts. Let us hypothetically assume that a speaker provides the following enunciation:

*I love my son above all other persons. On the other hand, I despise my ex-wife with the whole strength of my soul*.

Each voter was asked to assign emotions to the entire enunciation, which clearly expresses at least two different emotions. For the first part—*I love my son above all other persons*.—the emotion “love” is present. However, in the second part of the enunciation—*On the other hand, I despise my ex-wife with the whole strength of my soul*.—it is difficult to identify the emotion. It could be “anger”, “disapproval”, or “disappointment.”

The voters made the assignments independently of each other, and in this specific case, it is reasonable for them to cast different votes. The Common User algorithm harmonizes the discrepancies.

As the objective of this study is to establish a relationship, if one exists, between emotions and the Lacanian discourses, the combination of emotions is more representative of the Lacanian discourses adopted by the speaker and provides greater differentiation than single emotions. It is not an objective of this study to provide any diagnosis. In future studies, we will evaluate the possibility of using the Lacanian discourses as an auxiliary tool to be incorporated into diagnostic procedures.

The heat map shown in Figure 2 must be read row-wise, meaning that comparisons of values in the same row are meaningful. However, comparisons of values in different rows have qualitative meaning but not quantitative meaning. In the sequence, some examples will clarify these comments.

Out of the 67 rows of the heat map **only 7 have more than one value different from zero**. This result indicates that the identification of emotions has a very strong ability to identify the prevalent discourse in a sentence. The rows that have more than one relation intensity value different from zero are the following:

- {admiration, approval, excitement} **RI** {M, H, U} = 0.05;
- {admiration, approval, excitement} **RI** {H} = 0.82.
  It can be observed that the Hysteric discourse is identified in both instances. The relation intensity for the only Hysteric discourse is very strong. This is not surprising, as excitement is present in both sets of emotions. The presence of the Hysteric discourse along the Master and University may also occur, and this identification is certainly due to the *judgement* emotions—admiration and approval.
- {approval, caring, optimism} **RI** {M, A} = 0.07;
- {approval, caring, optimism} **RI** {A} = 0.13.
  It is reasonable to find the feeling of caring associated with the Analyst discourse. In a pure Analyst position, neither any kind of *judgment* nor *expectation* should appear, so the presence of approval and optimism counts toward the identification of the Master discourse as well.
- {approval, neutral} **RI** {M} = 0.38;
- {approval, neutral} **RI** {U} = 0.22.

and,

- {approval, realization} **RI** {M} = 0.36;
- {approval, realization} **RI** {U} = 0.39.
  In both cases, there is an ambiguity between the Master's and the University's discourses. The approval emotion is always present, i.e., the characteristic of *judgement* associated with these discourses. The other two emotions, neutral and realization, indicate the objectivity of these discourses. It is reasonable that such ambiguity occurs because **S1** is, in both cases, on the transmitter side, either as the *Agent* or the hidden *Truth*. Furthermore, **S1** either drives **S2** or addresses it.
- {caring, curiosity} **RI** {M, H} = 0.33;
- {caring, curiosity} **RI** {A} = 0.11.

and,

- {caring, curiosity, neutral} **RI** {H} = 0.54;
- {caring, curiosity, neutral} **RI** {A} = 0.09.
  In all cases, the emotions of caring and curiosity are present. They are distinctly associated with the Analyst discourse, but in a subtle and elusive way. This is probably why the intensity of the relation is low. However, caring and curiosity are not exclusive to the Analyst discourse. In the Hysteric discourse, the speaker may appear intensely, emotionally charged, seeking objective information (neutrality). In the Master discourse, the speaker may also show curiosity and care about the potential production of the receiver.
- {neutral} **RI** {M} = 0.04;
- {neutral} **RI** {U} = 0.78.
  Neutrality is expected from the speaker providing objective information, as in the case of University discourse. In a much lesser degree, neutrality may characterize a Master discourse when it describes a scenario in which the receiver is expected to act. Neutrality may be less pronounced in this case because the Master discourse is also characterized by authority, power, and self-image, which may imply biased speech.

The **only Master discourse** is uniquely identified by a very large set of emotion combinations, 10 in total. The relation intensity is in the range:


$$0.25\leq \left\{e_{i},e_{j},e_{k}\right\}\text{R}\text{I}\left\{M\right\}\leq 0.38,$$

and the emotions show a prevalence of judgement (approval, disapproval), self-image (pride), jouissance (joy), and expectation (anxiety, nervousness). This finding is consistent with **S1** in the position of *Agent / Semblance*, in which a role of power and authority may produce statements charged with judgement and expectation. On the other hand, **S1** driven by **$** speaks about himself in terms of the image he/she wants to display.

**The top four emotion combinations in relation to the Master discourse are:**

- {approval, neutral} **RI** {M} = 0.38;
- {approval, realization} **RI** {M} = 0.36;
- {neutral, pride} **RI** {M} = 0.33;
- {anxiety, nervousness} **RI** {M} = 0.27.

Several other emotion combinations exhibit the same relation intensity of 0.25.

The **only Analyst discourse** is uniquely identified by a set of five emotion combinations. The relation intensity is in the range:


$$0.08\leq \left\{e_{i},e_{j},e_{k}\right\}\text{R}\text{I}\left\{A\right\}\leq 0.20,$$

and the emotions show a prevalence of caring associated with curiosity and neutrality. This finding is consistent with **a** in the position of *Agent/Semblance* driven by **S2** in a role that shows empathy and a neutral curiosity to try to access the other's *Jouissance*. It is also worth noting that the numerical values of **RI** are consistently lower than in all other cases. This reflects the fact that in daily dialogues, people seldom adopt the neutral speech of an Analyst.

**The top five emotion combinations in relation to the Analyst discourse are:**

- {approval, caring, curiosity} **RI** {A} = 0.20;
- {approval, caring, optimism} **RI** {A} = 0.13;
- {caring, curiosity} **RI** {A} = 0.11;
- {caring, curiosity, neutral} **RI** {A} = 0.09;
- {caring, neutral, realization} **RI** {A} = 0.08.

**The only Hysteric discourse** is uniquely identified by a very large set of emotion combinations, 10 in total. The relation intensity is in the range:


$$0.33\leq \left\{e_{i},e_{j},e_{k}\right\}\text{R}\text{I}\left\{H\right\}\leq 0.82,$$

and the emotions span a wide spectrum, with excitement being prevalent. This finding is consistent with **$** in the position of *Agent/Semblance*, driven by **a** in a role that looks forward to gaining access to knowledge while expressing the power of personal feelings. It is worth noting that the numerical values of **RI** are consistently higher than in all other cases. This reflects how often, in daily life, people eagerly seek information and understanding, and also how this position is easier to be identified.

**The top five emotion combinations in relation to the Hysteric discourse are:**

- {admiration, approval, excitement} **RI** {H} = 0.82;
- {approval, curiosity} **RI** {H} = 0.75;
- {disappointment, sadness} **RI** {H} = 0.60;
- {anxiety, curiosity, nervousness} **RI** {H} = 0.50;
- {anxiety, disapproval, nervousness} **RI** {H} = 0.50.

**The only University discourse** is uniquely identified by a set of 8 emotions combinations. The relation intensity is in the range:


$$0.20\leq \left\{e_{i},e_{j},e_{k}\right\}\text{R}\text{I}\left\{U\right\}\leq 0.40,$$

and the emotions are characterized by neutrality, realization, and judgment. This finding is consistent with **S2** in the position of *Agent/Semblance*, driven by **S1** in a role that appears restricted to dealing with objective facts while simultaneously seeking the fulfillment of institutional objectives (**S2** is driven by **S1**) and employing judgment to achieve such results. It is worth noting that the numerical values of **RI** are quite similar to those obtained for the Master discourse.

**The top 5 emotions combinations in relation to the University discourse are:**

- {neutral} **RI** {U} = 0.78;
- {confusion, neutral, realization} **RI** {U} = 0.40;
- {approval, realization} **RI** {U} = 0.39;
- {annoyance, neutral} **RI** {U} = 0.27;
- {joy, neutral} **RI** {U} = 0.27.

**The only Capitalist discourse** is uniquely identified by a single combination of emotions, namely (approval, desire, joy). The relation intensity is 0.20. It is likely that if the experiment is applied to a larger set of dialogues, other combinations of emotions can be found. Nevertheless, this set of emotions well represents the fact that **$** in the position of the *Agent/Semblance* attempts to satisfy his/her internal needs not by addressing the *other* but by directly accessing an “artifact” that represents his/her desire, would produce joy, would have his/her approval, or would generate approval for him/herself, for example, in terms of “likes” on social media.

Many statements present a mix of Lacanian discourses. For example, **the combination {Master, Hysteric}** has appeared in 8 instances, and the relation intensity falls within the following range:


$$0.26\leq \left\{e_{i},e_{j},e_{k}\right\}\text{R}\text{I}\left\{M,H\right\}\leq 0.89,$$

and the top five emotion combinations in relation to this mix of discourses are:

- {annoyance, disappointment, disapproval} **RI** {M, H} = 0.89;
- {anxiety, realization} **RI** {M, H} = 0.64;
- {admiration, joy, surprise} **RI** {M, H} = 0.50;
- {annoyance, anxiety, embarrassment} **RI** {M, H} = 0.50;
- {caring, disappointment, embarrassment} **RI** {M, H} = 0.50.

**The combination {Hysteric, University}** has appeared in 5 instances, and the relation intensity is in the range:


$$0.25\leq \left\{e_{i},e_{j},e_{k}\right\}\text{R}\text{I}\left\{H,U\right\}\leq 0.55,$$

and the emotions combinations in relation to this mix of discourses are:

- {annoyance, disapproval, realization} **RI** {M, H} = 0.55;
- {confusion, disapproval, surprise} **RI** {M, H} = 0.50;
- {curiosity, desire, neutral} **RI** {M, H} = 0.50;
- {approval, disappointment} **RI** {M, H} = 0.29;
- {approval, joy, realization} **RI** {M, H} = 0.25.

**The combination {Master, University}** has appeared in three instances, and the relation intensity is in the range:


$$0.13\leq \left\{e_{i},e_{j},e_{k}\right\}\text{R}\text{I}\left\{M,U\right\}\leq 0.71,$$

and the emotional combinations in relation to this mix of discourses are:

- {approval, excitement, and pride} **RI** {M, U} = 0.71;
- {annoyance, anxiety, and disapproval} **RI** {M, U} = 0.40;
- {admiration, neutral, and realization} **RI** {M, U} = 0.13.

It is also worth noting that, for example, Anger—a **Basic Emotion** in the GoEmotions dataset—has not appeared. At this stage, a possible explanation is that the set of dialogues used in the experiment does not include this emotion, but it may appear in a larger experiment. Another possibility is that Anger may have been annotated as Annoyance or Disapproval. Another case is Grief—a **Secondary Cognitive Emotion** in the GoEmotions dataset—which has not appeared as well. In this case, a possible explanation is that Grief has been annotated as Sadness.

It is left to the interested reader to examine the heat map and verify that the set of emotions associated with the combination of discourses is consistent with Lacan's theory.

### 4.4 Sample size stability analysis

As detailed in Section 4.1, our experimental procedure considers a total of 264 sentences across 40 dialogues. We will now rigorously estimate the necessary sample size (i.e., the number of sentences) to guarantee a high level of precision for the examined probability of co-occurrence of combinations of discourses with emotions.

As noted, we estimate the proportion of sentences that satisfy a given property; for example, we examine the case in which discourse *d*_1_ occurs together with emotion *e*_1_. The necessary sample size *n* to guarantee a certain precision is given by


$$n\geq \frac{Z^{2}p\left(1-p\right)}{E^{2}}$$

where Z the z-score for the desired confidence level, *p* the expected (unknown) proportion and E the desired error margin.

For a worst-case sample size estimate, we assume the unknown probability *p* equals 1/2, so *p*(1 − *p*) is maximized. At a 90% confidence level (which we consider quite adequate for this challenging research setting, which inherently includes high subjectivity), *Z* = 1.645. For an error margin *E* = 0.05, we have:


$$n\geq \frac{{\left(1.645\right)}^{2}\cdot \frac{1}{2}\cdot \frac{1}{2}}{{\left(0.05\right)}^{2}}=270.60$$

Thus, our sample size of 264 sentences is very close to the required size of 270 sentences, making it adequate for the purpose of our analysis. In fact, for 264 sentences, the margin of error is E = 0.0506.

For additional experimental evidence in Section 24, we present the detailed findings of a meticulous stability analysis via simulation.

### 4.5 Detailed simulation study for sample size stability analysis

To complement the above analysis, we performed a very detailed experimental procedure.

#### (a) Probability convergence as sample size increases

We examine the following 26 sample sizes *s*_*i*_, 1 ≤ *i* ≤ 26 where sample size *s*_*i*_ = 10·*i* sentences. In other words, the studied sample sizes are (*s*_1_, *s*_2_, …, *s*_26_) = (10, 20, …, 260).

For each sample size *i*, we estimate all probabilities of discourses-emotions co-occurrence experimentally. To do so, we select 1,000 times a random subset of 264si sentences; each time we estimate the probability (according to Equation 4) and take the mean of the 1,000 estimated probabilities as the discourse-emotion probability *p*_*i*_ when the sample size is *s*_*i*_. This way, we get 26 different probability estimates (*p*_*i*_), each one corresponding to a sample size *s*_*i*_ (1 ≤ *i* ≤ 26).

**Figure 3:**
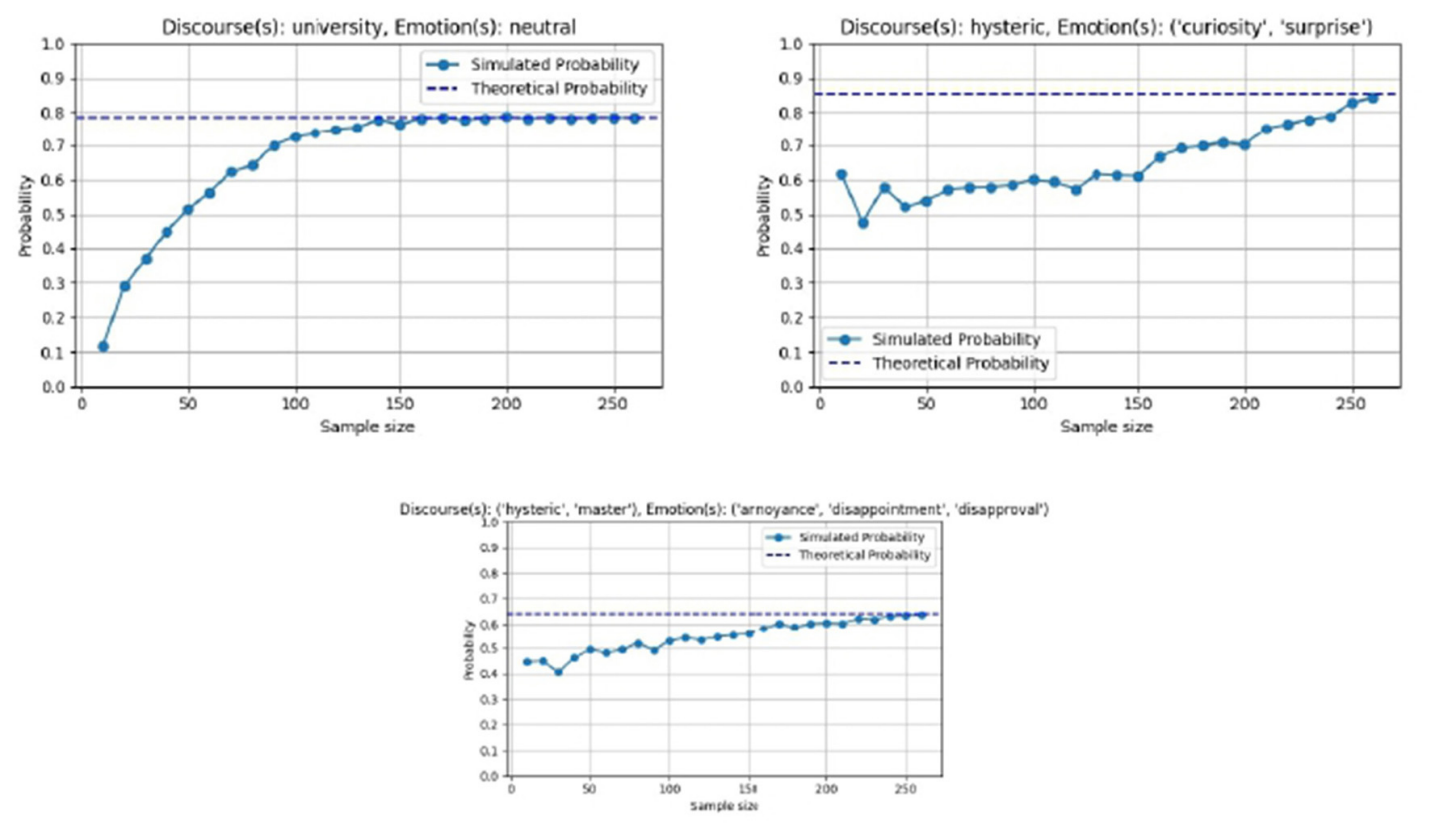
Convergence of the simulated probability to the theoretical probability for different numbers of discourses and emotions. **(Top left)** The case for one discourse (“University”) and one emotion (“Neutral”). **(Top right)** The case for one discourse (“Hysteric”) and two emotions (“Curiosity” and “Surprise”). **(Bottom)** The case for two discourses (“Hysteric” and “Master”) and three emotions (“Annoyance,” “Disappointment,” and “Disproval”).

In all cases, the simulated probability converges to the theoretical probability as the sample size increases. To illustrate, we provide three characteristic examples in Figure 3: one discourse and one emotion, one discourse and two emotions, and two discourses and three emotions.

#### (b) Mean value of estimated probability

For each discourse-emotion co-occurrence, we also estimate the mean μ of the 26 probabilities for this co-occurrence, each one corresponding to a sample size *s*_*i*_ (1 ≤ *i* ≤ 26). This will be used in the standard relative error analysis.

#### (c) Standard deviation estimation

We estimate the standard deviation (std) of these 26 probabilities *p*_*i*_, each one corresponding to a sample size *s*_*i*_ (1 ≤ *i* ≤ 26). For completeness, we estimate two different types of standard deviation: the cumulative standard deviation and the rolling standard deviation.

#### (c.1) The cumulative standard deviation

The cumulative standard deviation is as follows:


$$\begin{array}{l} c_{1}=0\\ c_{2}=\text{std}\left(p_{1},p_{2}\right)\\ c_{3}=\text{std}\left(p_{1},p_{2},p_{3}\right)\\ \vdots \\ c_{26}=\text{std}\left(p_{1},p_{2},\ldots ,p_{26}\right) \end{array}$$

**Figure 4:**
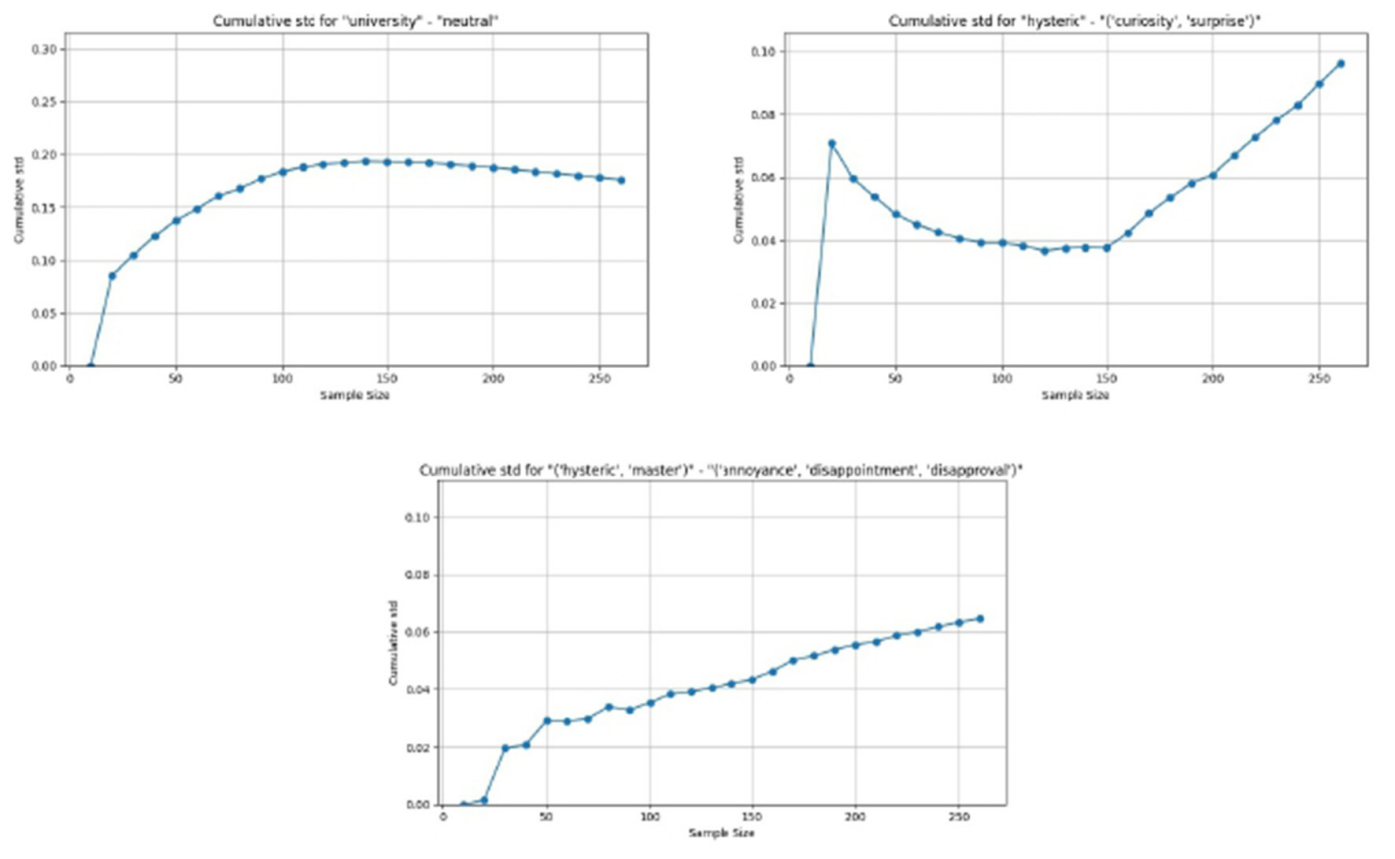
Convergence of the cumulative standard deviation for different numbers of discourses and emotions. **(Top left)** The case for one discourse (“University”) and one emotion (“Neutral”). **(Top right)** The case for one discourse (“Hysteric”) and two emotions (“Curiosity” and “Surprise”). **(Bottom)** The case for two discourses (“Hysteric” and “Master”) and three emotions (“Annoyance,” “Disappointment,” and “Disproval”).

In all cases, the standard deviation converges to a very low value as the sample size increases. We provide the findings for the three examples in Figure 4.

#### (c.2) The rolling standard deviation

The rolling standard deviation is defined as follows:


$$\begin{array}{l} r_{1}=\text{std}\left(p_{1},p_{2},\ldots ,p_{10}\right)\\ r_{2}=\text{std}\left(p_{2},p_{3},\ldots ,p_{11}\right)\\ \vdots \\ r_{17}=\text{std}\left(p_{17},p_{18},\ldots ,p_{26}\right) \end{array}$$

**Figure 5:**
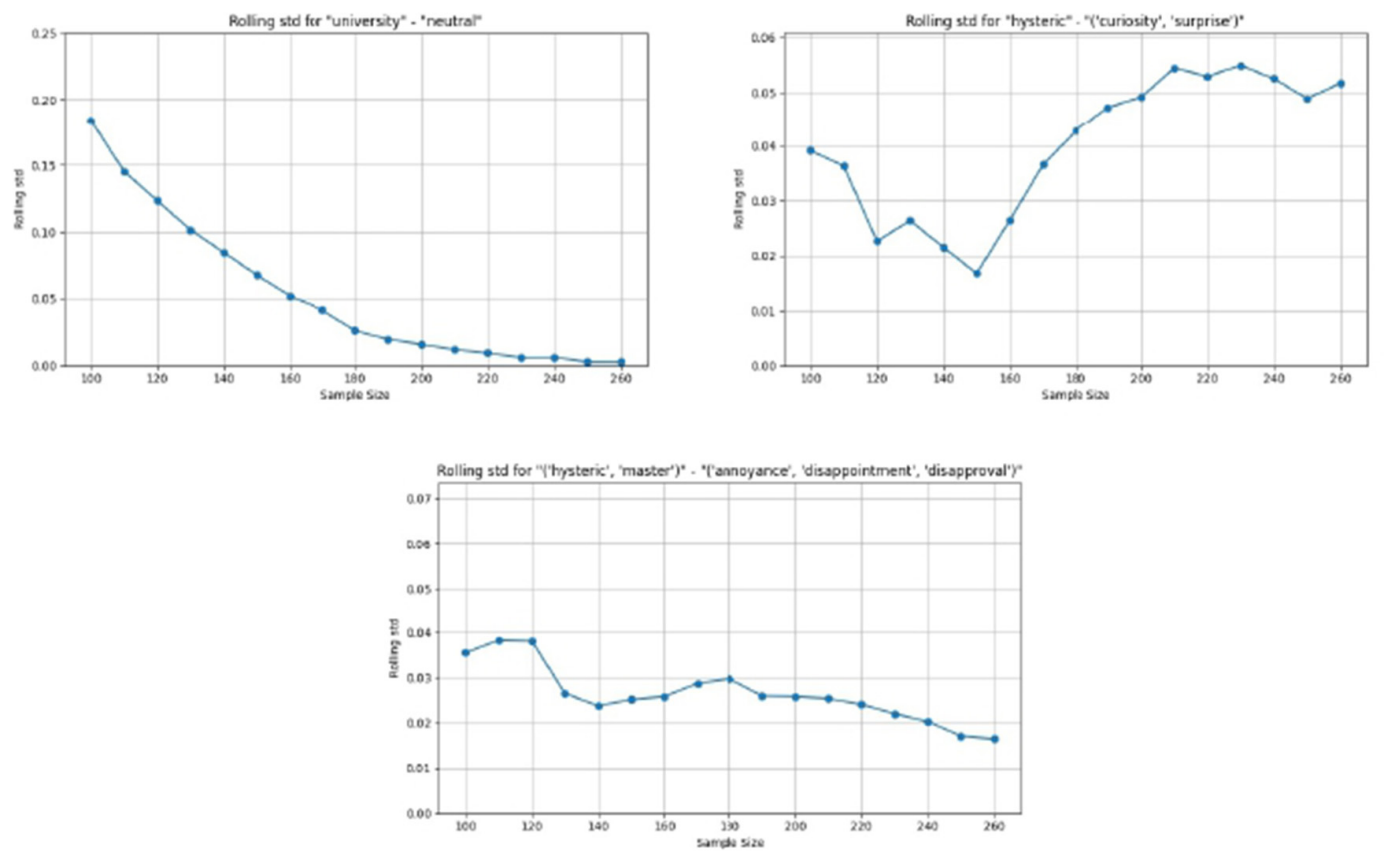
Convergence of the rolling standard deviation for different numbers of discourses and emotions. **(Top left)** The case for one discourse (“University”) and one emotion (“Neutral”). **(Top right)** The case for one discourse (“Hysteric”) and two emotions (“Curiosity” and “Surprise”). **(Bottom)** The case for two discourses (“Hysteric” and “Master”) and three emotions (“Annoyance,” “Disappointment,” and “Disproval”).

In all cases, the rolling standard deviations decrease fast with sample size, converging to a very low value. The findings for our three characteristic examples are shown in Figure 5.

#### (d) Relative Standard Error

Finally, we estimate the Relative Standard Error (RSE), defined as follows:


$$\text{RSE}=\frac{\text{std}}{\sqrt{n}\cdot \mu }$$

where std is the standard deviation, *n* is the sample size (1 ≤ *n* ≤ 264), and μ is the average of the probabilities *p*_*i*_ mentioned above. We estimate two versions of RSE: one for cumulative std and another for rolling std.

In the literature, the following regimes of RSE have been empirically associated to corresponding sample stability levels:


$$\begin{array}{l} \text{RSE}<1\%\text{ high stability}\\ \text{RSE}<5\%\text{ good stability}\\ \text{RSE}<10\%\text{ adequate stability} \end{array}$$

**Figure 6:**
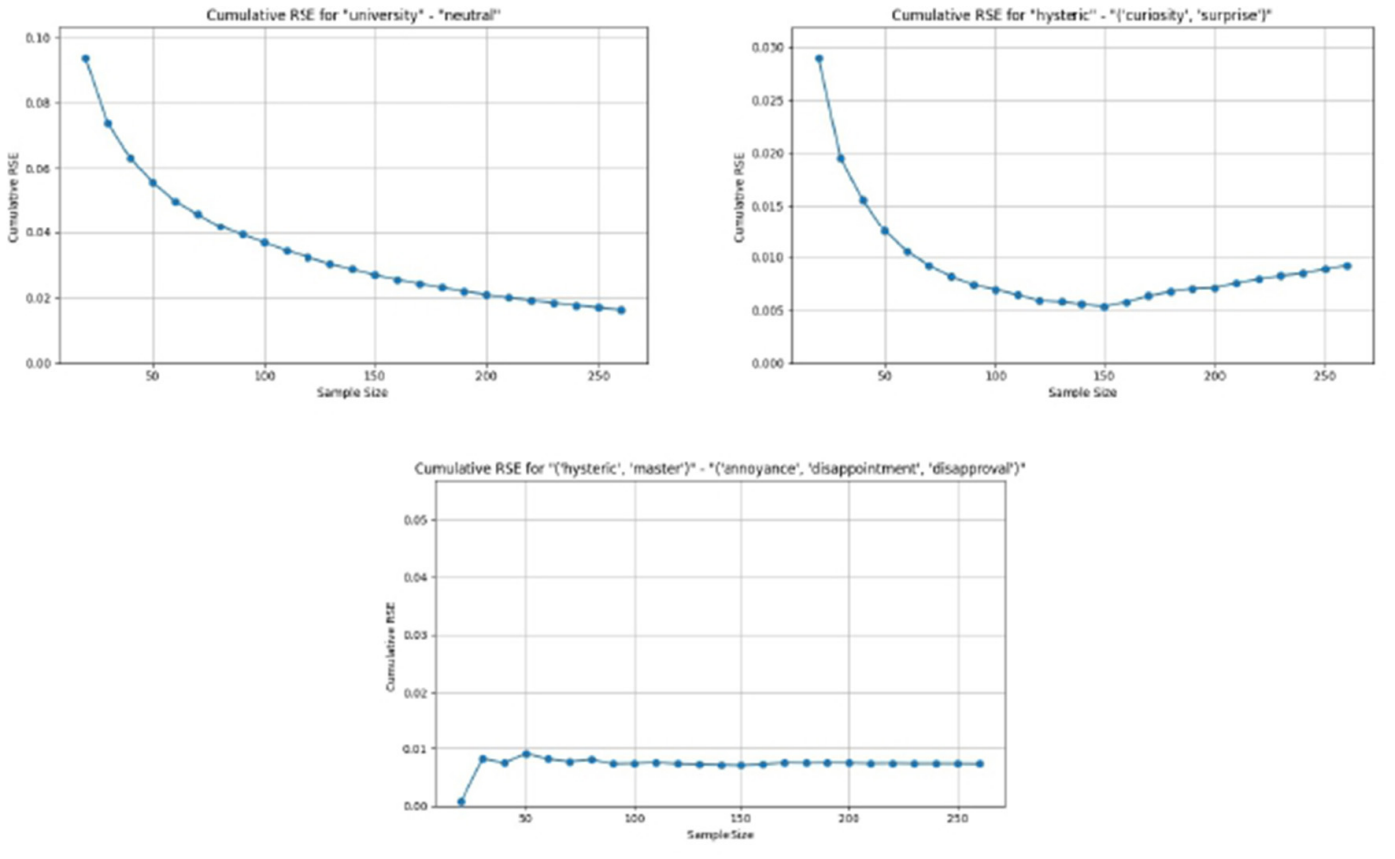
Relative Standard Error (RSE) based on the cumulative standard deviation for different numbers of discourses and emotions. **(Top left)** The case for one discourse (“University”) and one emotion (“Neutral”). **(Top right)** The case for one discourse (“Hysteric”) and two emotions (“Curiosity” and “Surprise”). **(Bottom)** The case for two discourses (“Hysteric” and “Master”) and three emotions (“Annoyance,” “Disappointment,” and “Disproval”).

**Figure 7:**
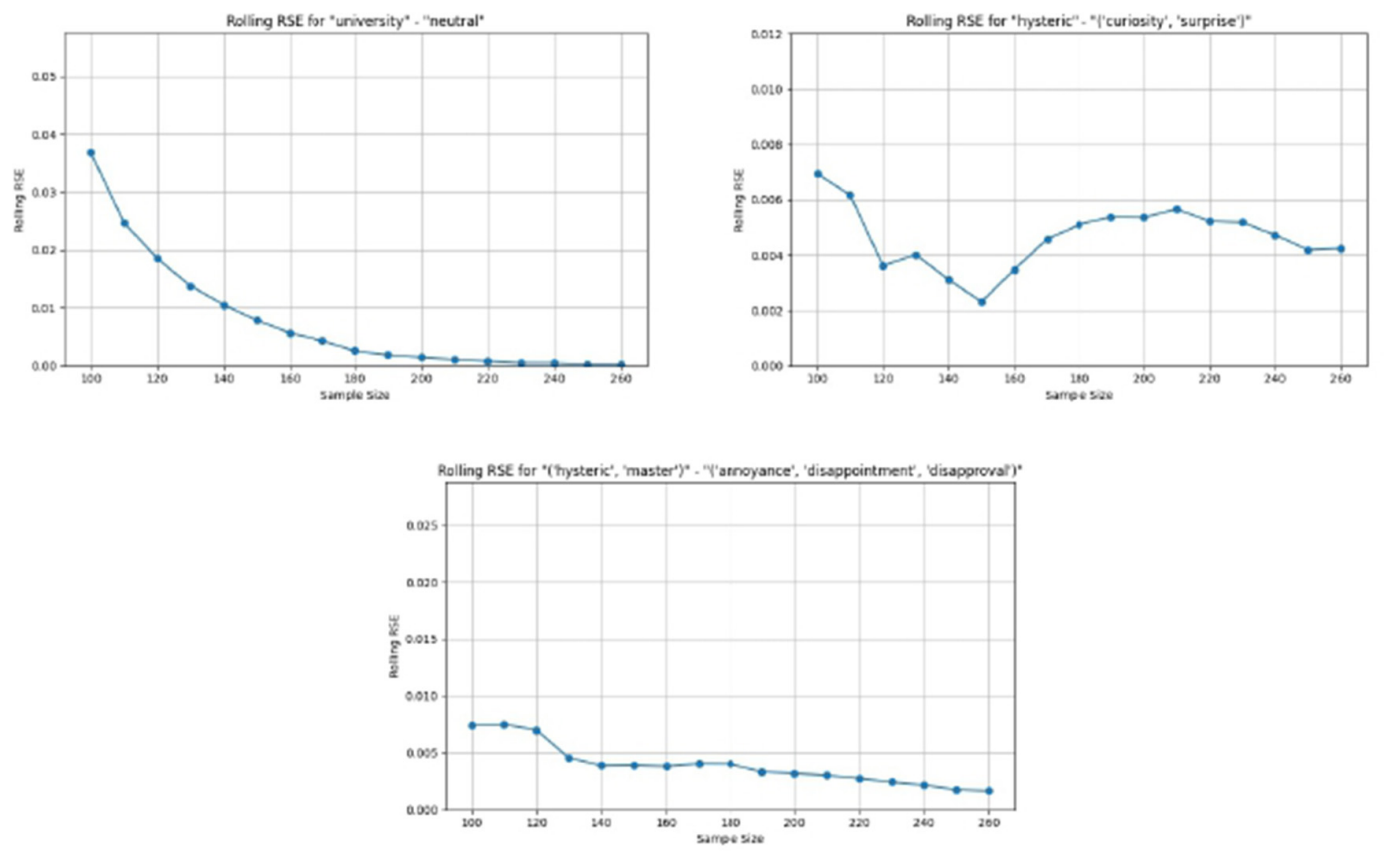
Relative Standard Error (RSE) based on the rolling standard deviation for different numbers of discourses and emotions. **(Top left)** The case for one discourse (“University”) and one emotion (“Neutral”). **(Top right)** The case for one discourse (“Hysteric”) and two emotions (“Curiosity” and “Surprise”). **(Bottom)** The case for two discourses (“Hysteric” and “Master”) and three emotions (“Annoyance,” “Disappointment,” and “Disproval”).

Notably, in all cases, the RSE is very small, consistently indicating high sample stability. We illustrate this for our three characteristic cases, showing the RSE calculated using both the cumulative standard deviation (Figure 6) and the rolling standard deviation (Figure 7).

The above findings experimentally indicate a quite high sample size stability.

## 5 Conclusions and future studies

The main contribution of this research is a psychoanalytic one per se: the establishment of a systematic relation among emotions and Lacanian discourses.

The main findings of this work can be summarized as follows:

- An empirical evidence of the relationship between the combination of emotions and Lacanian Discourses has been established;
- Emotions have a strong differential power to identify unique Lacanian discourses present in texts;
- Emotions also have a strong differential power to identify a mix of Lacanian discourses present in texts;
- The relationships identified can be explained psychoanalytically.

The findings of this work are limited by the small set of dialogues used in the experimental procedure. However, the procedure has proved to be powerful and can be applied to a larger set to refine the results. We conjecture that new relationships will be found and that those already found will be confirmed.

As shown in Section 4, we symbolized the relation intensity for particular combinations of discourses with a particular combination of emotions in the following way:


$$\left\{e_{i},e_{j},e_{k}\right\}\text{R}\text{I}\left\{d_{a},d_{b},d_{c}\right\}=r_{m},$$

where 0 ≤ {*i, j, k*} ≤ 30. The value 0 is used to represent “no emotion considered” and the other values point to a specific emotion of the dataset.

{*a, b, c*} represent one of the five Lacanian discourses or none of them to take into account a single or a combination of discourses.

We intend to improve the {*e*_*i*_, *e*_*j*_, *e*_*k*_}**RI**{*d*_*a*_, *d*_*b*_, *d*_*c*_} model. To do so, we will:

- Evaluate {*e*_*i*_, *e*_*j*_, *e*_*k*_}**RI**{*d*_*a*_, *d*_*b*_, *d*_*c*_} = *r*_*m*_ and establish the heat maps for each individual voter.
- Establish a new “average user” heat map based on the average value of the heat maps for each individual voter.
- Compare the models obtained for each individual voter, the “common user” and the “average user” and refine the conclusions obtained so far.
- Open the platform to students and other researchers to collect more votes and improve the model.

Regarding future study, it is worth noting that this LDD method can be automated to a great extent, since current computer-based methods (primarily employing AI systems and tools) can effectively detect emotions in texts. Narratives will be used as input to the MentaLLaMA (Yang et al., 2024), the first open-source instruction-following LLM series for interpretable mental health analysis on social media. The detected emotions and their combinations will feed our LDD model to identify the Lacanian Discourses.

This way, there is great potential to develop effective, real-world applications based on the automated identification of emotions and corresponding discourses.

In Mota et al. (2012) and Mota et al. (2014), the authors describe a speech-graph-based quantitative measure of thought disorder in psychosis. The method described in those papers has been implemented by Psychomeasure (https://psychomeasure.com), which, according to its website, is a European start-up dedicated to transforming mental health through innovation in Healthtech and Digital Therapeutics. With this tool, doctors can track their patients' progress remotely and identify meaningful changes in their mental health. This platform enables earlier interventions, personalized care, and a significant leap toward transforming mental health management.

The graph corresponding to a given narrative is independent of the language and semantics. This graph can be annotated with the results obtained by the LDD. In this way, clusters of nodes will be assigned to specific discourses. This new and enhanced graph representation could potentially provide:

- a deeper understanding of the narrative;
- an improved binary classification of mental disorders; and
- a transdiagnostic process identification.

This study is just the first step of the Lacanian Discourse Discovery (LDD) methodology, which is presented for the first time here. In the future, a structural approach will be developed to directly identify the signifiers **S1, S2, $, and a** based on their unique properties, without relying on emotions as an intermediate step.

## Data Availability

The datasets generated and analyzed during this study are available on GitHub at the following link: https://github.com/PsyComp-Psychoanalytic-Driven-Computing/dailydialog_lacanian_discourses_emotions.

## Footnotes

1 NLP is not to be confused with Neuro-Linguistic Programming Therapy.
